# Advances in applied supramolecular technologies 2021–2025†

**DOI:** 10.1039/d4cs01037j

**Published:** 2025-09-04

**Authors:** Dominick E. Balderston, Elba Feo, Anamaria Leonescu, Mackenzie Stevens, Alexander M. Wilmshurst, Philip A. Gale, Cally J. E. Haynes, George T. Williams, Jennifer R. Hiscock

**Affiliations:** a University of Kent Canterbury CT2 7NH UK J.R.Hiscock@Kent.ac.uk; b School of Physical and Mathematical Sciences, Faculty of Science, University of Technology Sydney Ultimo 2007 NSW Australia philip.gale@uts.edu.au; c Department of Chemistry, University College London 20 Gordon Street London WC1H 0AJ UK cally.haynes@ucl.ac.uk; d School of Chemistry and Chemical Engineering, University of Southampton Southampton SO17 1BJ UK G.T.Williams@soton.ac.uk

## Abstract

Supramolecular chemistry is a rapidly evolving field that has focused on building a foundation of fundamental understanding in controlling molecular self-assembly, through the use of non-covalent interactions. A common criticism of the field is that whilst the systems produced are very elegant, they do not have real-world use. Therefore, focus is now moving to applying the fundamental understanding of supramolecular chemistry to the production of commercially viable products. Building on our previous review in this area, which described the translational potential of innovations within the field of supramolecular chemistry up to the year 2020, we now review the progress of this field over the years 2021–2025 with the aim to inspire researchers to apply supramolecular chemistry to solve real world problems, moving innovation out of the laboratory and into the commercial marketplace.

## Abstract 2

Over the last five years the field of supramolecular chemistry has demonstrated exceptional translational potential with an explosion in successfully commercialised technologies, alongside a healthy innovation pipeline.

## Graphical abstract

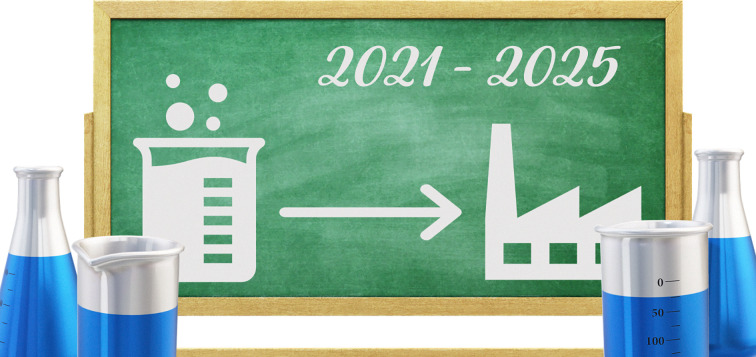

## Introduction

Chemistry is often referred to as the ‘central science’ due to its wide-reaching influence on other fields including medicine, biology and engineering. Supramolecular chemistry, defined as ‘chemistry beyond the molecule’, studies how molecular species associate due to the formation of non-covalent interactions, including those with partial covalent character.^1^ Since the term ‘supramolecular’ was first coined in 1978,^2^ there have been major advances in both fundamental understanding and the application of concepts associated with this subfield of chemistry, resulting in the development of a wide range of systems with properties applicable to a wide range of industrial sectors.

In this review, we provide an overview of a range of technologies, all underpinned by supramolecular concepts, that offer commercial potential or have already achieved translational success, illustrated with commercial case studies. This builds upon previous reviews in this area by Kolesnichenko and Anslyn (2017),^3^ Smith and co-workers (2025),^4^ as well as one by authors of this review (2017–2020),^5^ to provide an overview of those translational advances made within supramolecular chemistry between 2021–2025 across multiple commercial sectors.

### Methodology

In constructing this review, we opted for a combination of a peer-reviewed journal led and patent/web lead approach to defining the literature included within the scope of this article. It is because of the translational, real-world application of the science to be discussed that we have let the patent and commercialisation of supramolecular innovation confirm or lead our searches for peer-reviewed literature.

#### Peer-reviewed journal led approach to literature searching

This workflow was developed to make conventional strategies for journal literature searching relevant to the patent literature. Initially a conventional literature search was undertaken, and articles that were application driven in the relevant field of interest were identified. From these articles the corresponding author names were noted.

This information was then used to search for any patents on which the corresponding authors were named as inventors or owners of a patent, through the searching of patent databases such as Espacenet^6^ and google patents.^7^ Of these databases, Espacenet was found to be the most effective as it covers 97 countries, whereas Google Patents only covered 17 countries at the time of writing. A useful feature of these databases is the effective translation of patents, increasing the accessibility of these texts.

A limitation of this method of searching is that for common names, this process can return a lot of results, so we also added date restrictions to our search criteria. With any patents relating to the journal manuscript of interest identified, the contents of the patent were examined to make sure that they contained the same or related content to that detailed within the journal article. If the journal article and the patent content was found to correlate, then the journal article was included within the scope of this review.

#### Patent-based approach to literature searching

As there can be some discrepancy between corresponding author names on a journal article and the inventor or ‘owner’ of a patent, based on the ownership of intellectual property rights, a patent first workflow was also developed to identify relevant translational supramolecular chemistry innovations.

In contrast to the approach described previously, here an initial search was first made using field specific keywords within the patent database Espacenet.^6^ The results from this keyword search were then refined by date. Where multiple patents were found to be related, these entries were grouped and classed as a single ‘hit’. Where peer-reviewed journal articles were directly linked within the patent filing, the content of these articles was used within this review. Where no peer-reviewed journal article was directly referenced within the patent filing, a search of peer-reviewed journal articles was made using keywords, inventor and patent owner data supplied by the patent. Where no peer-reviewed journal article could be found linked to a patent filing, the data communicated within the patent was not included within this review.

#### Searching the web for evidence of translational innovation

We acknowledge that in some cases there may be no patent filing related to a translational supramolecular innovation. Therefore, an internet search was conducted using openly accessible search engines and related keywords to identify any companies that have successfully managed to translate a supramolecular innovation within the 2021–2025 timeframe. Where evidence of supramolecular commercialisation was identified, information supplied on a company website was used to search for peer-reviewed journal articles, that were then included within the scope of this review.

### Supramolecular innovation in everyday things

#### Supramolecular chemistry in the household

**Fig. 1:**
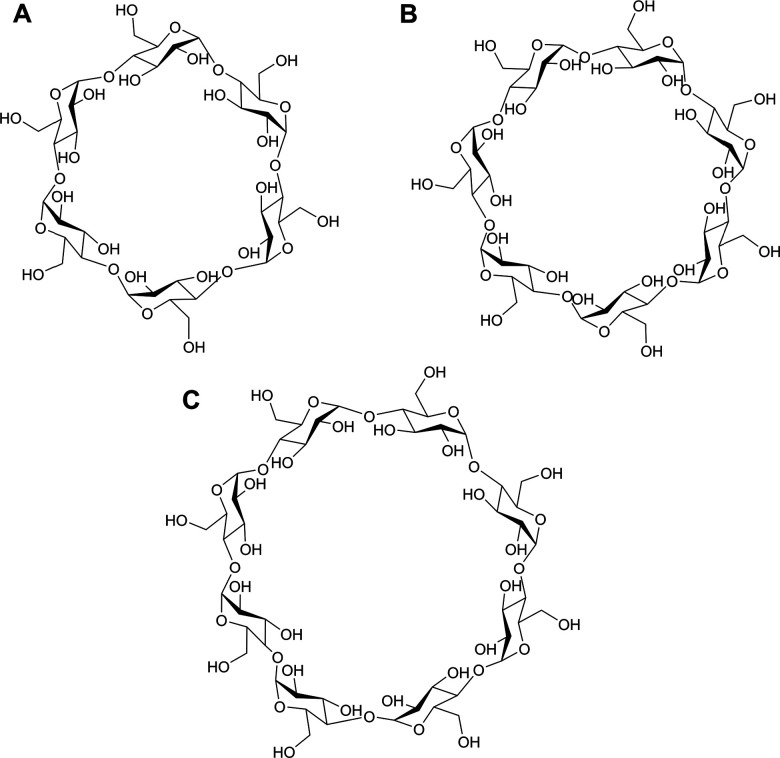
Chemical structures of (A) α-CD (B) β-CD and (C) γ-CD.

Supramolecular chemistry is fundamental to the function of many everyday products including those commonly found in the household. For instance, cyclodextrins (CDs) are a class of macrocyclic supramolecular host that can be found in many dermopharmaceutical and cosmetic products, such as sun creams, shampoos, deodorants, fragrances and acne creams.^8,9^ CDs are cyclic oligosaccharides, linked by 1–4 glycosidic bonds consisting of a hydrophobic central cavity and a hydrophilic outer surface, which can accommodate small hydrophobic drugs and other moieties.^10,11^ CDs are manufactured on an industrial scale from starch, with the commonly available CDs being α-CD, β-CD, and γ-CD, which have 6, 7, and 8 d-glucose units respectively (Fig. 1). β-CD is the most widely used in the cosmetics industry due to its ease of production and low price.^10^ However, β-CD has low solubility, limiting use in some applications. Therefore, modified β-CD derivatives such as the hydrophilic hydroxypropyl-β-cyclodextrin (HPBCD) have been developed to remove this limitation by increasing solubility. CDs can form host–guest complexes with non-polar guest molecules, resulting in improved solubility and stability under aqueous conditions, as well as enabling controlled release into the skin.^12^ In addition, free CDs are used in shampoos and deodorants to capture sebum and odour molecules, binding them to the interior of the cavity.^8^ The use of CDs for dermopharmaceutical and cosmetic applications has been recently reviewed by Paiva-Santos and co-workers.^8^

CDs have also been developed to provide novel solutions that enable water purification. The presence of organic micropollutants, such as pesticides, in water resources is a growing concern due to the adverse health effects that they can cause.^13^ Activated carbon is the most common material used to remove organic pollutants, however there is a need to develop more efficient materials capable of removing a greater range of micropollutants. To meet this need, Dichtel, Helbling and co-workers have developed porous β-CD-containing polymers (P-CDPs).^14^ By cross-linking β-CD with rigid aromatic molecules, a high-surface-area, mesoporous polymer was created, capable of capturing micropollutants within the hydrophobic CD cavity. P-CDP was shown to outperform Norit RO 0.8 activated carbon (the industrial standard) in the removal of a mixture of organic micropollutants at environmentally relevant concentrations. These pollutants included aromatics (1-naphthyl amine, 2-naphthol, 2,4-dichlorophenol), pesticides (metolachlor), plastic components (bisphenol A, bisphenol S) and pharmaceuticals (propranolol, ethynyl oestradiol).

##### Commercial case study: CycloPure

In 2016 Dichtel and Cassou founded the company CycloPure to commercialise P-CDPs.^353,354^ CycloPure now produces a variety of products containing the DEXSORB® material for both large-scale applications and for home use, including home filtration systems that plug into main water lines and countertop water jug filters. DEXSORB® is also capable of removing per- and polyfluoroalkyl substances (PFAS) from water.^15^ PFAS are a growing concern due to their persistence in water and adverse health effects.^16,17^ Following a series of pilot programs across the US, in 2024, the Massachusetts Department of Environmental Protection approved the use of DEXSORB® to remove PFAS from drinking water systems throughout the state.^18^ Additional supramolecular systems designed to remediate PFAS beyond the scope of the household are discussed in greater detail later in this review.

##### Commercial case study: AgroFresh

The use of 1-methylcylopropene (1-MCP) to prevent fruit and vegetable ripening was pioneered by Sisler and Blankenship in 1996.^19^ Ethylene is a growth regulator in plants that affects developmental processes including ripening. 1-MCP binds with ethylene receptors, supressing the ethylene response pathway and delaying ripening.^20^ The company AgroFresh was founded to commercialise the use of 1-MCP. The product SmartFresh™ uses a formulation of 1-MCP with cyclodextrin, resulting in a stable powder that can be dissolved in water and released as a gas for use on produce.^21^ The use of SmartFresh™ extends the shelf life of produce and reduces food waste throughout the supply chain.

**Fig. 2:**
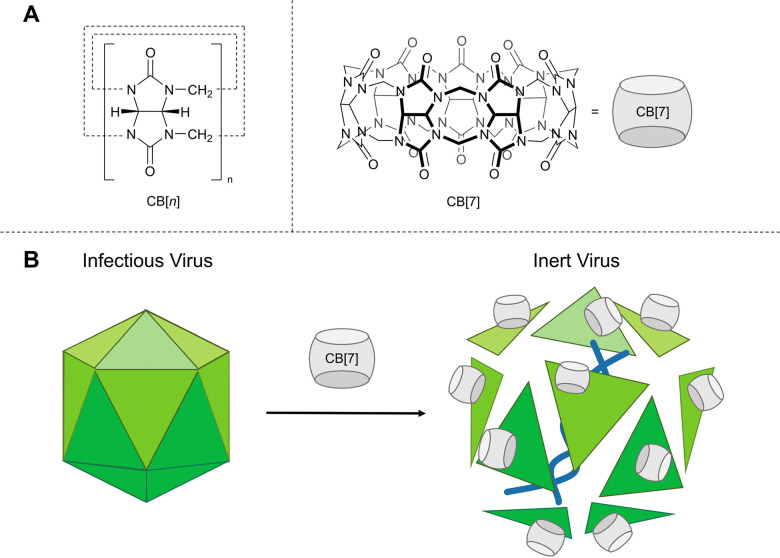
(A) Chemical structure of cucurbit[*n*]urils (CB). (B) Illustration depicting the virucidal antiviral effect of CB[7].^24^

Curcurbit[*n*]urils (CB) are another class of macrocycle, capable of acting as a supramolecular host molecule with demonstratable commercial application. The general structure of these macrocycles is shown in Fig. 2A. CBs are composed of “*n*” glycoluril monomers linked by methylene bridges to form barrel shaped macrocycles, that are capable of binding guest molecules within the hydrophobic cavity of the macrocycle.

##### Commercial case study: Aqdot®

The company Aqdot® uses the host–guest binding of CBs in their AqFresh™ odour control technology. Malodour and pollutant molecules are strongly bound into the cavity of the CB thus supressing odours.^22^ The company is now exploring the use of these compounds as antiviral disinfectants.^23^ Aqdot®, in collaboration with Samuel Jones, have demonstrated the virucidal activity of CBs, *via* host–guest complexation between the CB cavity and viral surface proteins (Fig. 2B).^24^ Initial tissue culture infectious dose assays with herpes simplex virus-2 (HSV-2) in Vero cells (kidney epithelial cells) showed CB[7] and CB[*n*] (a mixture containing *n* = 6, 7 and 8 in the ratio of 4 : 2 : 1) inhibit HSV-2. To confirm whether the antiviral mode of action was dependent on the formation of a CB : viral protein complex, a 1 : 1 complex of CB[7] and ferrocene was also tested for antiviral activity under analogous experimental conditions. Ferrocene binds strongly to the CB[7] cavity (*K*_a_ > 10^6^ M^−1^),^25^ meaning the cavity is occupied and unavailable for binding to the virus. The 1 : 1 complex of CB[7] and ferrocene showed no antiviral activity confirming that host: guest binding of the viral protein within the cavity of CB[7] is vital to antiviral properties.

To evaluate whether the CB[*n*] formulation met the European standard for disinfectants intended for use in a medical area, the standardised method EN 14476 was employed. The virucidal activity of the CB[*n*] formulation was established against poliovirus 1, murine norovirus, adenovirus type 5, modified vaccinia virus, and feline coronavirus, using a maximum of 5 mg mL^−1^ CB[*n*] to reflect the concentrations in commercially available CB-products. Murine norovirus, modified vaccinia virus, and feline coronavirus were shown to be susceptible to the CB[*n*] formulation, exhibiting approximately a 1-log reduction in viral titre at the highest tested concentration. Though this is a promising result, it is insufficient to reach the EN 14476 standard at present.

Finally, the standardised assay ISO 18184 was used to test CB[*n*] as a textile surface disinfectant. Unlike other virucides such as bleach, CBs are not damaging to surfaces and are not irritating to skin, marking them as good candidates for development as surface disinfectants. At 5 mg mL^−1^ application of the CB[*n*] formulation resulted in a 98.4% reduction in feline coronavirus when added onto cotton inoculated with the virus, and a 99% reduction in murine norovirus. When the textile was pretreated with the CB[*n*] formulation, a 90% reduction in viral titre was achieved against murine norovirus. On polyester-lycra, post inoculation treatment with CB[*n*] resulted in a 62% reduction of murine norovirus and 90% reduction of feline coronavirus. These results indicate that CBs are promising virucidal agents with applications as soft surface disinfectants.

Cellulose and chitin are polysaccharides that provide structural support within plants (cellulose), fungi and arthropods (chitin).^26^ These polysaccharides are often present as fibrils which can be arranged in different ways to create hierarchical structures. Such hierarchical structures can lead to ‘structural colour’, a phenomenon that is caused by the reflection of light from the periodic ordering of the fibrils. This can lead to various optical effects due to the polarisation of light created due to the presence of chiral helicoidal structures. These effects can be observed in the vivid, metallic-like shells of certain insects and skin of fruit, such as the *Pollia* fruit.^27^ Structural colour has benefits over traditional absorption-based pigments. As the colour is a result of the periodic structure, the entire visible spectrum can be accessed through control of self-assembled morphologies and photobleaching does not occur.^26^

**Fig. 3:**
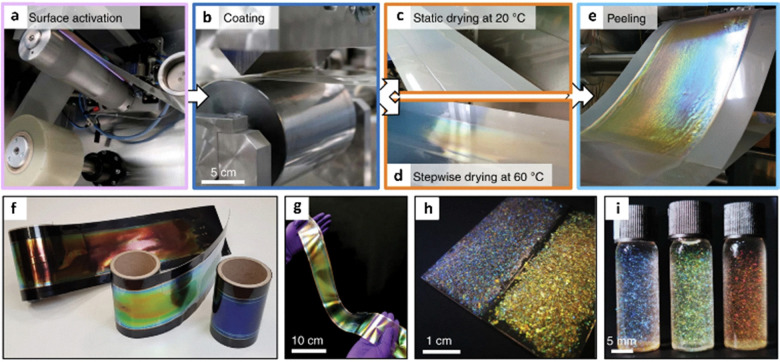
Overview of the roll-to-roll (R2R) production of photonic films from cellulose nanocrystals (CNC) (A) activation of the polymer substrate *via* corona etching. (B) Deposition of CNC suspension onto a polymer substrate. (C) Static drying of the CNC film at room temperature. (D) Stepwise hot air drying of the CNC film. (E) Peeling of the CNC film from the polymer substrate. (F) Red, green, and blue CNC films on a black polymer substrate. (G) Free-standing CNC film. (H) Pristine (left) and heat-treated (right) photonic CNC particles in varnish. (I) Heat-treated photonic particles in ethanol (left), 50% aqueous ethanol (centre), and water (right). Reproduced from ref. 28 with permission from Springer Nature, copyright 2021.

To mimic this naturally occurring behaviour, cellulose nanocrystals have been used to form photonic films by solvent-evaporation-driven self-assembly. Vignolini, Parker, and co-workers developed have developed a method to produce large scale photonic films *via* roll-to-roll deposition.^28^ Here, an aqueous suspension of cellulose nanocrystals is deposited and dried on a polymer substrate. The film is then peeled from the polymer, resulting in a free-standing film. Subsequent, heat treating, grinding, and size-sorting results in particles that can be used as effect pigments and glitter (Fig. 3). The development of eco-friendly pigments is desirable to replace unsustainable inorganic pigments and microplastic glitters.

##### Commercial case study: Sparxell

Droguet and Vignolini co-founded the company Sparxell, which uses this cellulose nanocrystal technology to create vibrant, fade-resistant, biodegradable pigments that can be used for a range of applications including cosmetics, textiles, packaging, and paints.^29^ As cellulose is edible, the pigments can also be used in food and beverages. This has prompted further investigation into the applications of structurally coloured cellulose compounds for use in smart food packaging and labelling.^30,31^

#### Supramolecular chemistry for battery materials

Since the invention of the lithium-ion battery (LIB), batteries have become integral to enabling our everyday lives, powering everything from laptops and cell phones to electric vehicles. In 2019, Goodenough, Wittingham, and Yoshino were awarded the Nobel Prize in Chemistry for their contributions to the development of the lithium-ion battery.^32^ However, there is an increasing demand for safe, robust, low-cost, high-performance batteries for use in new technologies like electric vehicles and wearable electronics.^33,34^ Current LIBs have unstable flammable liquid electrolytes, which has led to the development of solid electrolytes to try and produce safer batteries with increased thermal and mechanical stability.^35^

Solid electrolytes need to have both high ionic conductivity and mechanical robustness. Polymers have received a lot of attention as potential solid electrolytes due to favourable physiochemical properties including high flexibility, and ease of processing.^33,36^ One of the challenges of polymer electrolytes is attaining a good ionic conductivity whilst maintaining good mechanical strength. One strategy to overcome this problem is to use block copolymers, where one block provides the ionic conductivity and the other the mechanical strength. However, these result in rigid electrolytes that are not compatible with applications requiring flexible and stretchable batteries, such as for healthcare devices, implantable devices, and artificial skin.^37,38^

##### Commercial case study: anthro energy

**Fig. 4:**
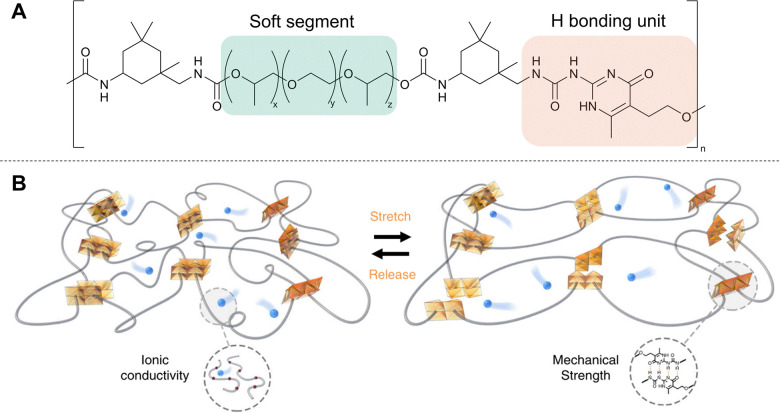
(A) Chemical structure of SLIC polymer. (B) Illustration depicting the effect of stretching the SLIC polymer. (B) Is reproduced from ref. 39 under creative commons license CC BY 4.0.

Mackanic and co- workers have been able to mitigate issues associated with rigidity through the design of a supramolecular lithium-ion conductor.^39^ The polymer consists of polyether backbone units to provide the ionic conductivity, and 2-ureido-4-pyrimidone (UPy) backbone units to provide mechanical strength (Fig. 4).^40^ Each UPy unit can form four hydrogen bonds to another unit forming a ‘dimer’. These hydrogen bonds can be broken to allow the material to stretch, with SLIC-3 showing an extensibility of ∼2700% ± 63% and an ultimate stress of 14 MPa ± 0.2 MPa. SLIC-3 also showed good stress recovery, with the (*n* = 1) polymer completely recovering its shape after a 1 h rest. The authors show that SLIC can be used as a binder material to make intrinsically stretchable LIB electrodes and go on to demonstrate how the SLIC electrodes and SLIC electrolyte can be combined to make a battery used to power a lightbulb that remains lit when the battery is stretched up to 70% strain and folded in half. This research resulted in the spin out of Anthro Energy in 2021 to commercialise this technology.^41,42^

**Fig. 5:**
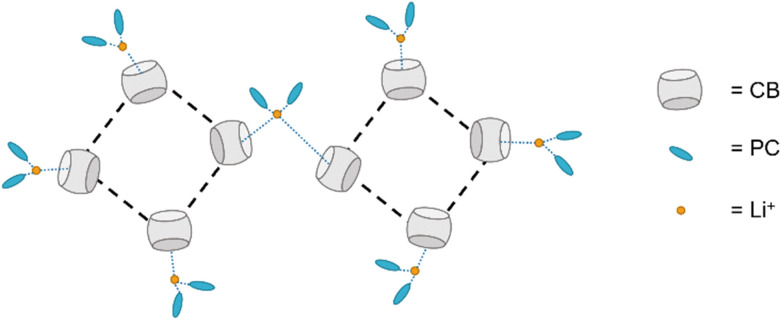
Diagram depicting the intermolecular interactions between CB, PC, and Li^+^ ions within the electrolyte.^43^

Taking a different approach, Xie and co-workers have designed a CB[6] (Fig. 2A) incorporated supramolecular electrolyte for use in lithium metal batteries (LMBs).^43^ This electrolyte combines CB[6], LiClO_4_ and propylene carbonate (Fig. 5). The hydrogen bonding between CBs provides a flexible framework and the Lewis acid–base interactions between CB[6], while the partially solvated lithium ions facilitate fast ion transportation through the structure. The electrolyte exhibits a bulk Li^+^ conductivity of 2.9 × 10^−4^ S cm^−1^ at 25 °C with a low Li^+^ diffusion activation energy of 0.29 eV.

Moving away from LIBs, sodium ion batteries (SIBs) are actively being explored as a potential alternative due to their comparable low cost of manufacture and the environmental sustainability of sodium.^44^ However, one of the challenges of SIBs is the large volume change that occurs during sodiation/desodiation cycles which causes particle pulverisation of the electrodes leading to rapid capacity decay on repeated cycling. This is because sodium cations are larger than lithium cations.

**Fig. 6:**
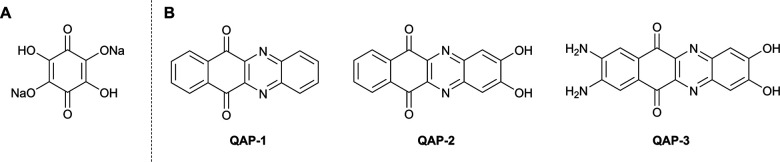
(A) Chemical structure of tetrahydroxy-1,4-benzoquinone disodium salt^45^ and (B) quinone-fused aza-phenazines 1–3.^46^

A potential solution to this limitation associated with the development and use of SIBs is the incorporation of supramolecular self-healing electrode materials. Huang and co-workers employed a tetrahydroxy-1,4-benzoquinone disodium salt (Fig. 6A) as an organic cathode, where the intermolecular hydrogen bonding between hydroxyl and carbonyl groups is able to ‘heal’ cracks in the material.^45^ The long-term cyclic stability of the self-healing material was examined at high current densities of 1 A g^−1^ and 2 A g^−1^, indicating a long cycle life of 1000 cycles with negligible capacity loss.

As an alternative to traditional battery technologies, organic electrode materials (OEMs) represent a metal free, environmentally friendly alternative to the use of metal oxide and metal phosphate cathodes in LIBs. To support the development of these materials, Zhang and co-workers have established a correlation between the electrical performance of OEMs and the addition of functional groups that allow hydrogen bonding and π–π stacking.^46^ For this purpose they chose a series of quinone-fused aza-phenazines (QAPs) with different hydrogen bonding donor and acceptor motifs (Fig. 6B). The compound with the greatest number of hydrogen bond donors (QAP-3) displayed the best electrical properties, with the highest discharge capacity and capacity retention. This is attributed to the synergistic π–π stacking and strong hydrogen bonds in QAP-3 resulting in a closely packed structure. Crystal structures indicate that QAP-1 has longer weak C–H⋯N_Ar_ interactions (2.682 and 2.668 Å), whereas in QAP-3 π-stacked columns are connected *via* alternating O–H⋯N_Ar_ (1.847 Å), O–H⋯O=C (2.041 Å), N–H⋯O=C (2.227 Å), and N–H⋯OH (1.946 Å) interactions. This study shows that non-covalent interactions can be leveraged to design OEMs with increased stability and performance.

#### Supramolecular chemistry for electronics

**Fig. 7:**
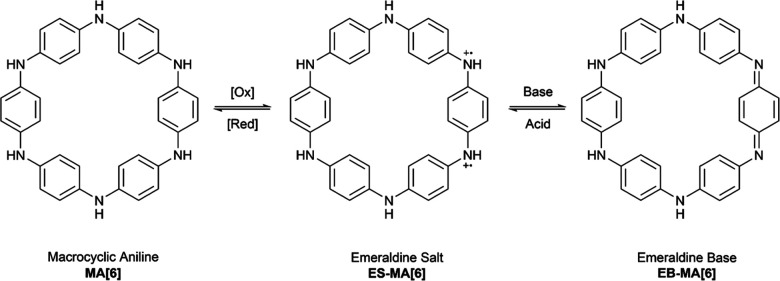
Chemical structure of the macrocyclic aniline MA[6], as well as the emeraldine salt and emeraldine base forms.^52^

Materials capable of both ionic and electrical conductivity are desirable for many applications including chemical sensors,^47^ bioelectronic probes,^48^ and electrochemical transistors for circuits and neuromorphic computing.^49–51^ Organic mixed ionic-electronic conductors (OMIECs) have emerged as ideal materials for these applications due to their processability.^49^ Nuckolls, Evans, and co-workers have synthesised a hexameric macrocyclic aniline (MA[6]), that can be converted to its ‘emeraldine salt’ form (ES-MA[6]) by reaction with trifluoroacetic acid (TFA), in the presence of oxygen (Fig. 7).^52^ The doubly oxidised ES-MA[6] displays pH dependent electrical and ionic conductivity. Crystals were produced by slow evaporation of heptane into 5% TFA/acetonitrile and solid zinc, resulting in ES-MA[6] co-crystalised with Zn[CF_3_CO_2_]_2_. X-ray diffraction indicates the positive charges are equally distributed among the six nitrogens of the macrocycle. ES-MA[6] assembles into trimers stabilised by π–π stacking and hydrogen bonding with TFA. These interactions lead to the formation of long-range nanotubes. The formation of nanowires in ES-MA[6] thin films was confirmed with powder X-ray diffraction, atomic force microscopy, and scanning electron microscopy. To assess the conductivity of the material, devices with spin-cast films of ES-MA[6] on Si/SiO_2_, and Ag or Au contacts were made. Exposing an Au contact thin film device to a 1.0 mM solution of NH_3_ resulted in an over 400-fold decrease in the conductivity. After subsequent exposure to 1.0 M TFA full recovery of the conductivity was observed. The process was shown to be reversable over at least seven cycles. The switchable and reversible electrical conductivity of MA[6] indicates potential applications in chemical detectors or neuromorphic computing.

#### Supramolecular chemistry in solar cell materials

To reduce greenhouse gas emissions, global electricity generation must move away from fossil fuels towards environmentally friendly and renewable alternatives such as solar cells. Power generation from solar cells tripled from 2018 to 2023 and accounted for 5.4% of global electricity generation in 2023.^53^ Reducing the cost of electricity from solar cells is key to further growth in this sector, and improving the efficiency of solar cells is one of the main challenges.

**Fig. 8:**
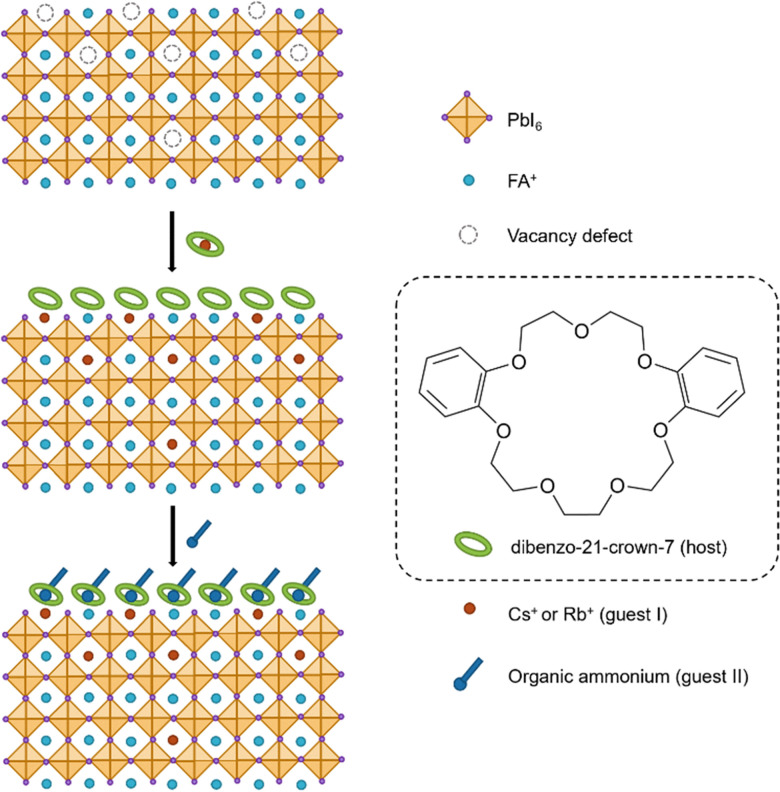
Schematic of the supramolecular perovskite interface and the dual host–guest complexation strategy.^59^

Perovskite solar cells (PSCs) have gained a lot of attention due to their optoelectronic properties, low cost of materials, and simple fabrication methods.^54^ Perovskite solar cells have developed significantly in the last 15 years with the first studies in 2009 showing power conversion efficiency (PCE) of 3.8%^55^ to a current certified value of 27.0%.^56^ One of the main challenges to the commercialisation of PSCs is the instability of the interface between the perovskite and carrier transport layers. This means the lifetimes of PSCs are not yet at the level of current silicon based solar cells. One strategy that has been employed to improve the performance of PSCs is the use of supramolecular compounds to reduce the concentrations of perovskite defects.^57,58^ Zhao and co-workers recently demonstrated that a dual host–guest (DHG) complexation strategy can be used to modulate the properties of FAPbI_3_-rich perovskites.^59^ The perovskite surface is first treated with a caesium salt and a crown ether (dibenzo-21-crown-7) (Fig. 8), resulting in Cs^+^ doping of the bulk perovskite and passivation of defects. Then the modified perovskite is treated with phenylethylammonium iodide. The phenylethylammonium cation forms a complex with the crown ether and acts as a passivating layer that minimises charge recombination. The DHG treated PSC had a certified PCE of 25.5% and retained 96.6% of its initial efficiency after 1050 hours at 25 °C. The authors attribute the performance and stability of the devices to the supramolecular interactions between the ammonium and crown ether at the interface of the perovskite and hole conductor layers. This work provides a promising strategy for improving the performance of PSCs.

#### Molecular solar thermal energy systems incorporating supramolecular chemistry

Whilst there have been great strides in both conventional silicon and perovskite-based photovoltaics, these technologies convert solar energy into electricity, which requires storage in batteries that, as previously discussed, can come at great environmental costs. Molecular solar thermal (MOST) energy systems instead use molecular photoswitches to convert light energy from the sun into stored chemical energy, which can readily be released as heat. Heating and cooling accounts for around half of final energy use globally, so alternative technologies able to convert, store and supply solar energy as heat offer an ideal way to tackle climate goals.^60^

**Fig. 9:**
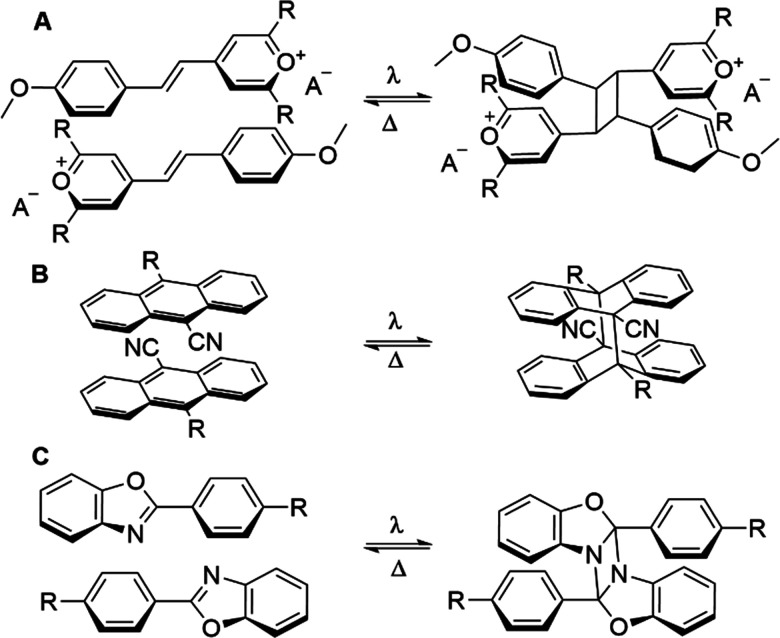
Photoswitches developed by Han and co-workers which are orientated by supramolecular interactions to enable efficient cycoadditions: (A) styrylpyrilliums,^63^ (B) 9,10-di-substituted anthracenes^64^ and (C) *p*-functionalised phenylbenzoxazoles.^65^

Upon irradiation a photoswitch is converted into a metastable state of a higher energy; upon back conversion from the metastable to stable state this energy difference is released. The majority of switches used in MOST systems do not rely on supramolecular effects, and we point the reader to previously published works.^61^ Additional applications of molecular photoswitches are also outlined later in this review. However, unlike these unimolecular MOST systems, Han and co-workers have utilised supramolecular interactions to promote efficient solar energy storage in biomolecular systems, with a focus on reversible photochemical cycloadditions. The first example published by the group surrounded the use of styrylpyrillium salts (Fig. 9A);^62^ these compounds feature donor–acceptor character and thus orientate in a head-to-tail fashion in the solid state. These favourable π–π interactions enable facile photo-triggered [2+2] cycloaddition in the solid state on exposure to 400–600 nm light, forming a strained four-membered ring resulting in energy storage densities of >50 J g^−1^. Building upon their styrylpyrillium work, these authors moved towards the investigation of systems capable of [4+4] cycloadditions in the solid state, namely donor–acceptor anthracene compounds.^63^ By incorporation of an electron withdrawing and electron donating group at the 9- and 10-positions respectively these compounds mirror the styrylpyrillium salts in their head-to-tail arrangement in the solid state, with red shifted absorbance compared to unmodified anthracene (Fig. 9B). These anthracene dimers were capable of now storing >200 J g^−1^, with enough energy release to self-activate. To demonstrate this, the authors showed that a solid pellet of anthracene dimer could be activated by local heating with a laser, and the heat produced by the [4+4] cycloreversion is capable of a cascade activation process, triggering the bulk material to reach a temperature of 165 °C. The most recent investigation from Han and co-workers outlines the use of *p*-functionalized phenylbenzoxazoles which undergo [2+2] photocycloadditions to form diazetidine adducts.^64^ These compounds display a remarkable energy storage of >300 J g^−1^, owing to the dearomatisation that occurs (Fig. 9C). These seminal examples represent a novel strategy towards converting solar into chemical energy, whilst also storing it without the need for conventional batteries.^65^

### Applied supramolecular innovations in medicine

#### Intracellular glucose sensing

Glucose homeostasis is tightly regulated through both hormonal and neural mechanisms that maintain blood glucose levels within a narrow physiological range.^66^ In healthy individuals, this regulation is a pivotal process which ensures a consistent glucose flux to meet the required metabolic demands of the body, whilst also maintaining overall functional stability.^66,67^ Major fluctuations or disruptions in blood glucose levels are associated with increased incidence of diseases such as cancer, Alzheimer's, and diabetes.^68–70^

Diabetes is a major, long-term medical problem, and remains among the top 10 causes of death in adults, associated with an estimated 4 million deaths globally in 2017.^71^ According to the international diabetes federation report (2021), an estimated 537 million people were currently affected by diabetes, with these numbers projected to rise to 643 million by 2030.^71,72^ Of these cases, ∼10% of individuals are type 1 diabetics (inability to produce insulin) and the remaining 90% are type 2 (no sensitivity to insulin).^71,72^ Although both type 1 and 2 diabetes can be managed through insulin therapy, issues with this approach remain. For instance, fluctuations in blood glucose levels often require frequent adjustments to maintain glycaemic control. However, this dynamic dosing can inadvertently lead to incorrect levels of insulin being administered, resulting in inordinately low glucose levels (hypoglycaemia), which can be life-threatening.^73,74^ Alternatively, not injecting enough insulin could also have long-term implications if blood glucose levels remain too high (hyperglycaemia), such as peripheral neuropathy, cancer, or cardiovascular diseases.^75–77^

The clinical significance of glucose in disease incidence and the challenges associated with insulin therapy highlights the necessity for accurate mechanisms to detect and monitor internal glucose levels. Common strategies that enable internal glucose detection employ glucose oxidase.^78^ However, as an enzymatic probe, the activity of glucose oxidase can be easily affected by environmental factors, and is subject to degradation *in vivo*.^79,80^ The development of small-molecule fluorescent dyes has also been proposed as a means to circumvent these limitations, such as 18F-FDG, a radiotracer used in PET scans for tumour detection.^81^ However, while these probes are capable of indirectly monitoring glucose levels through changes in glucose uptake, they fail to directly monitor intracellular glucose levels.

**Fig. 10:**
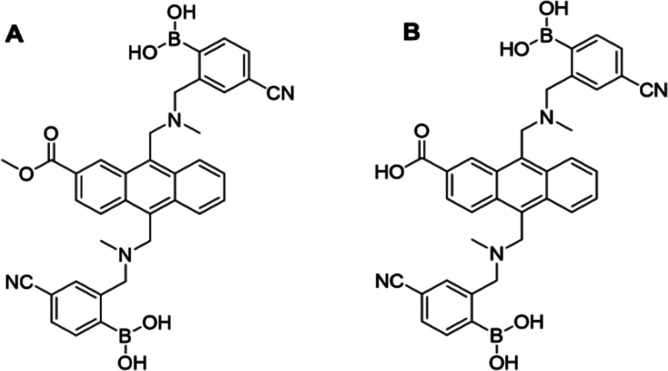
Structure of boronic based probes (A) Mc-CDBA and (B) Ca-CDBA.^84^

Work by Shinkai and co-workers in 1995 led to the synthesis of a fluorescent probe, phenyl(di)boronic acid (PDBA), with a high selectivity for glucose *versus* other monosaccharides.^82^ While PDBA maintained the ability to directly bind to glucose under physiological conditions, this probe required the addition of an organic solvent to aid solubility, preventing use in biological systems.^82^ Furthermore, intracellular glucose levels often vary within the micromolar range, requiring the enhancement of PDBA sensitivity to enable accurate intracellular glucose level imaging.^83^ However, subsequent developments by James and co-workers has led to the development of two boronic acid-based fluorescent PDBA probes, Mc-CDBA and Ca-CDBA; which incorporated a cyano group *para* to a boronic acid functionality on the benzene ring system (Fig. 10).^84–86^ The addition of the cyano-substituent resulted in the greatly enhanced sensitivity of Mc-CDBA probe to glucose, when compared to PDBA alone (*F*/*F*_0_ = 47.8, 0.1 M glucose and *F*/*F*_0_ ≈ 14.5, 0.1 M glucose respectively).^84^

**Fig. 11:**
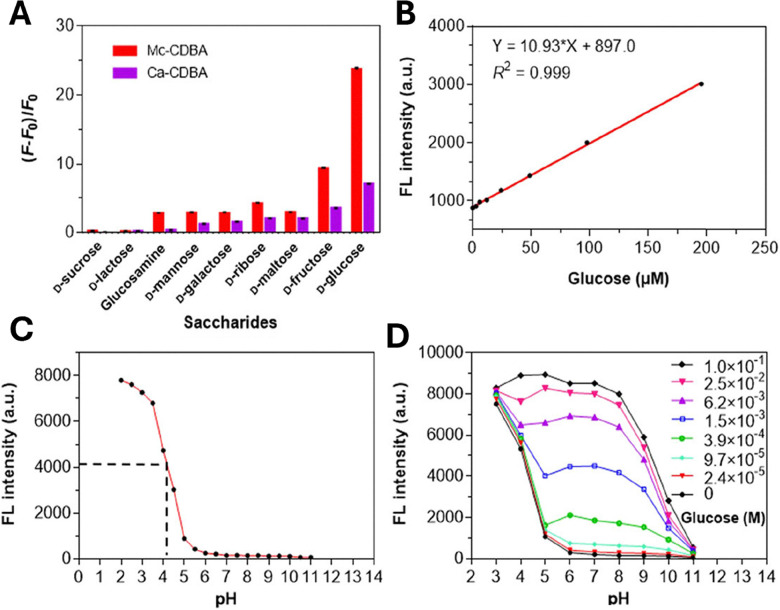
Fluorescence and glucose sensitivity of Ca-CDBA and Mc-CDBA (A) fluorescence response (*F* − *F*_0_)/*F*_0_) of Mc-CDBA and Ca-CDBA to alternate saccharides at a 1.56 mM concentration. (B) Linear fluorescence response of Mc-CDBA (10 μM) to increasing glucose concentrations (0–195 μM). (C) pH dependent fluorescence intensity of 10 μM Mc-CDBA in PBS buffer. (D) pH dependent fluorescence intensity of 10 μM Mc-CDBA in various glucose concentrations (0–0.1 M). Measurements of both Mc-CDBA and Ca-CDBA were analysed in 0.5% MeOH/PBS buffer and 0.5% DMSO/PBS buffer, respectively, pH = 7.4 at 25 °C (Mc-CDBA, *λ*_ex/em_ = 393/457 nm; Ca-CDBA, *λ*_ex/em_ = 382/438 nm). Data are presented as the means ± SD (*n* = 3). Reproduced from ref. 84 with permission from Journal of the American Chemical Society copyright 2023.

Kinetic experiments confirmed the capabilities of Mc-CDBA and Ca-CDBA to rapidly detect glucose with a good photosensitivity under physiologically relevant conditions. Furthermore, both probes exhibited a selective fluorescence response towards glucose over other saccharides such as d-fructose and glucosamine (Fig. 11) that was retained over a pH range of 5–8.^84^ A linear corelation between fluorescence intensity and glucose concentration was observed, with a biologically relevant detection limit for both Mc-CDBA and Ca-CDBA in a multitude of biological systems, such as blood plasma (48.8 μM to 12.5 mM and 24.4 μM to 12.5 mM respectively), interstitial fluid, sweat, and saliva (Fig. 11B and C).^84,87–90^

**Fig. 12:**
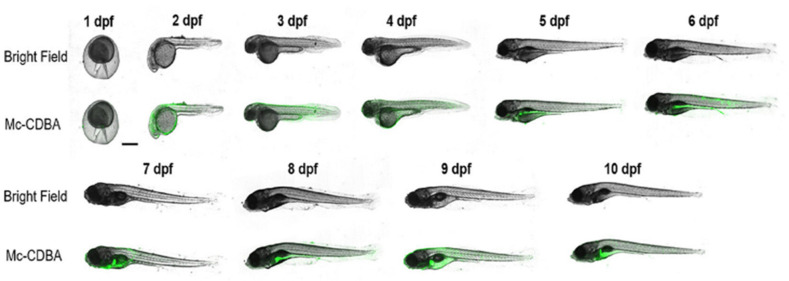
Confocal microscopy images of zebrafish embryos (1–10 days post fertilisation) incubated for 3 hours with 50 μM Mc-CDBA. Reproduced from ref. 84 with permission from journal of the American Chemical Society copyright 2023.

To evaluate the *in vivo* applicability of both Mc-CDBA and Ca-CDBA, James and co-workers utilised transparent zebrafish embryos as biological models, due to their suitability for live imaging.^84^ Here, the primary aim of this experimental procedure was to assess the probes’ effectiveness in detecting endogenous glucose levels. The uptake and fluorescence intensity of Mc-CDBA far exceeded that exhibited by Ca-CDBA, illuminating after one hour and three hours respectively. However, further imaging studies revealed that physiological glucose levels in zebrafish embryos are inherently low during early development (1–4 days post-fertilisation), as shown in Fig. 12. However, at 5–10 days post fertilisation glucose is observed to be concentrated to the embryo's pancreas, liver and intestines, consistent with prior published findings and confirming the applicability of these next generation boronic acid-based glucose sensors.^84,91^

**Fig. 13:**
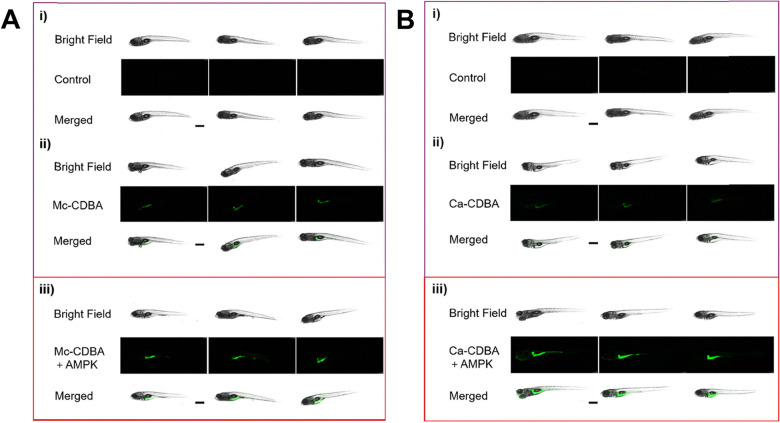
*In vivo* fluorescence imaging of Ca-CDBA and Mc-CDBA in zebrafish embryos. (A) Confocal fluorescence images in zebrafish embryos 7 days post-fertilisation pre-treated for 4 hours with a blank medium (i) and (ii), and 20 μM ampkinone (iii) followed by a 1-hour incubation with 50 μM Mc-CDBA (ii) and (iii). (B) Confocal fluorescence images in zebrafish embryos 7 days post-fertilisation pre-treated for 4 hours with a blank medium (i) and (ii), and 20 μM ampkinone (iii) followed by a 1-hour incubation with 50 μM Ca-CDBA (ii) and (iii). Imaging was performed using a Leica TCS SP8 confocal microscope (excitation at 405 nm; emission collected at 410–600 nm). Data are presented as mean values (*n* = 5); ****P < 0.0001. Scale bar = 500 μm. Reproduced from ref. 84 with permission from Journal of the American Chemical Society copyright 2023.

Moving beyond an application in glucose sensing, Mc-CDBA and Ca-CDBA were also employed as a mechanism of drug screening *in vivo*, demonstrated using the same zebrafish models. More specifically, for screening against ampkinone, an AMP protein kinase activator known to enhance glucose uptake and regulate blood pressure in diabetic individuals.^92^ Treatment with ampkinone led to a significant increase in fluorescence when compared to a control group where zebrafish were incubated with just Mc-CDBA and Ca-CDBA (Fig. 13). Conversely, treatment with the glucose transport inhibitor 4,6-EDG led to a slight reduction in fluorescence, though this was not statistically significant, attributed to high ambient glucose concentrations.^84^ When compared with alternative glucose sensors and enzyme probes aforementioned, the di-boronic acid probes presented by James and co-workers have the ability to report on intracellular glucose in multicellular biological models, due to their dynamic and reversible nature.

##### Commercial case study: Ziylo and Carbometrics

While boronic acid-based glucose sensors have demonstrated significant promise, they display disadvantages such as oxidative susceptibility.^93^ Alongside James and co-workers, Davis and co-workers, have made significant contributions to the advancement and successful translation of supramolecular receptors capable of successfully and selectively binding carbohydrates, with particular emphasis on glucose using a different supramolecular approach to molecular design.^94^

**Fig. 14:**
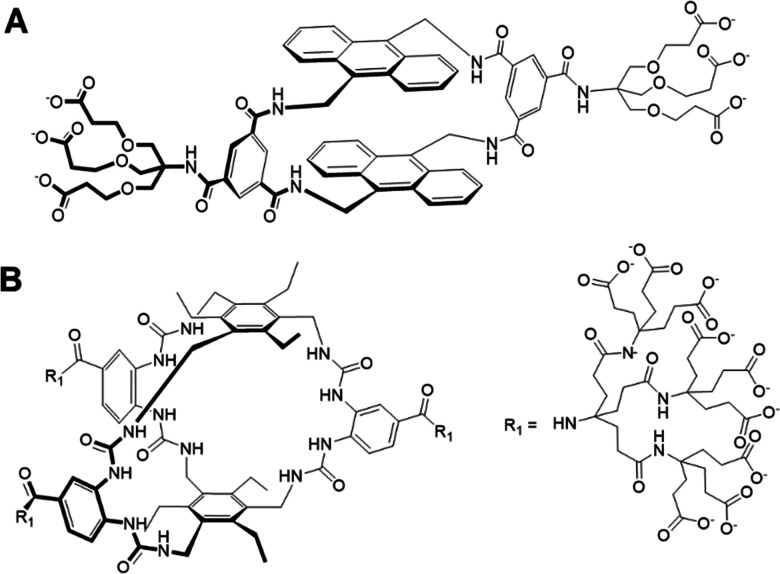
(A) The bis-anthracenyl macrocycle that achieved glucose binding in aqueous conditions (*K*_a_ = 60 M^−1^)^95^ and (B) the chemical structure of ‘GluHUT’.^98^

In 2012 Davis and co-workers produced a bis-anthracenyl macrocycle (Fig. 14A) which bound glucose under aqueous conditions, achieving an association constant of *K*_a_ = 60 M^−1^,^95^ detecting glucose within a relevant physiological range of ∼2–12 mM.^96^ Additionally, it was observed that fluorescence emission dramatically increased upon glucose binding, confirming the potential of this system to be developed as a glucose signalling system.^95,97^ This foundational work led to the creation of university spin-out company Ziylo.

Following the founding of Ziylo, Davis and co-workers continued in development of a more practical glucose receptor which demonstrated greater efficacy and selectivity. This was eventually achieved in October 2016, with “GluHUT” (Fig. 14B), a glucose receptor exhibiting roughly a 100-fold increase in glucose affinity when compared to previous molecular designs.^98,99^ This innovation was deemed as the key to filling the market gap, and the innovation was subsequently patented by Ziylo.^100^ In 2018, Ziylo concluded a deal with the world's major supplier of insulin, Novo Nordisk, worth around $800 million, highlighting the strategic value of GluHUT.^101^ As part of the arranged deal, the spin-out company Carbometrics was founded to work with Novo Nordisk on the development of glucose sensors. Carbometrics has continued to progress the development of glucose sensors with high optical output, selectivity and long-term stability, continuing to expand its commercial portfolio.^96^

Since this deal with Novo Nordisk, Carbometrics has continued to steadily advance its selective molecular glucose binding technology platform,^102^ and are now reported to be designing new polymeric materials, winning the Henry Royce Institute grant to develop novel polymers for optical glucose sensing.^103^ In addition, their work has also led to the development of a glucose-sensitive insulin (NNC2215), with an adjustable bioactivity to achieve better glycaemic control both *in vitro* and *in vivo*.^104^ NNC2215 was developed through the conjugation of a glucose binding macrocycle and a glucoside to insulin. In turn, they introduced a switch that can respond to glucose and equilibrate insulin between active and less active conformations.^104^ Furthermore, as of 2025, Carbometrics have developed a range of products and building blocks that have various *in vivo* applications, ranging from fluorescence microscopy to therapeutics. For instance, they offer ultra-stable red-emitting dyes that exhibit low protein binding for utilisation in advanced imaging techniques, alongside hydrolytically and oxidatively stable linkers, ensuring that dye attachment remains intact within harsh physiological conditions.^105^

Since Ziylo's collaboration with Novo Nordisk, Carbometrics has continued to forge strategic R&D partnerships. In May 2023, Carbometrics announced a research collaboration and option to license agreement with PyroScience GmbH, a German optical sensor company.^106^ This agreement aimed to create novel optical continuous-glucose-monitoring sensors for bioprocessing and cell-culture use, leveraging Carbometric's glucose binding molecules with PyroScience's sensor expertise.^106^ The UK's innovate UK/UKRI programme funded this effort *via* a UK-Germany grant awarded in September 2023, specifically to develop optical glucose sensors with PyroScience.^107^

#### Enhancing drug solubility and efficacy

A drug or drug candidate must exhibit an *in vivo* solubility high enough to elicit the desired therapeutic effect.^108^ A molecule, with limited *in vivo* solubility may demonstrate diminished biological activity, or have restricted administration routes.^108^ There are a number of ways to increase *in vivo* solubility, such as molecular structure optimisation, incorporation into a prodrug, salt formation, solubilising agent inclusion, CD complexation, and particle size reduction.^108^ Of these solubilisation methods, CD complexation is widely used throughout industry. In 2016 alone, 2200 peer reviewed journal articles and over 2300 patent submissions contained CDs, the majority of which relate to the pharmaceutical sector.^108^ As previously discussed, the presence of both hydrophobic and hydrophilic regions within the structure of CDs (Fig. 1), coupled with their truncated cone structure, facilitates the formation of host–guest complexes. The formation of drug–CD inclusion complexes, improves both a drug (candidate) photokinetic profile and therapeutic efficacy, hence CDs remain an important excipient to increase *in vivo* solubility.^109^

Due to these *in vivo* solubility enhancing properties, CD has also been used to enable drug repurposing. Irbesartan (IRB) and candesartan cilexetil (CAC) are traditionally used as hypertension drugs, which work as angiotensin II receptor blockers.^110^ By this same mode of action, IRB and CAC have been shown to reduce aqueous humour production, and as a result, reduce intraocular pressure (IOP). Therefore, IRB and CAC also demonstrate the potential to be repurposed as ophthalmic drugs, as confirmed through *in vivo* testing. However, low aqueous solubility and high lipophilicity has prevented this to date.^111^

To remove this limitation, Loftsson and co-workers have used γ-CD to complex IRB and CAC, both in the presence and absence of the water-soluble polymer Soluplus.^110^ The presence of Soluplus (a polyvinyl caprolactampolyvinyl acetate-polyethylene glycol graft co-polymer) increased the solubility of IRB and CAC in aqueous conditions by 55-fold and 228-fold respectively, when compared to the drug alone.^110^ The aqueous solubility of IRB and CAC further increased upon the addition of γ-CD. The amphiphilic nature of Soluplus, which exhibits a low critical micelle concentration (CMC) of 0.8 mg mL^−1^, promotes the encapsulation of poorly soluble drugs, however also decreases the 1 : 1 drug–CD complexation efficiency.^110^ Where drug–CD complex formation was not achieved, the Soluplus solubilised the remining drug into nanoaggregates, in addition to the drug–CD 1 : 1 complexes. This process was confirmed through DLS and zeta potential measurements, supported by TEM imaging. This discovery has led to several patent applications, relating to the formulation of drugs to be repurposed for ophthalmic use.^112–114^

#### *In vivo* applications of supramolecular materials

**Fig. 15:**
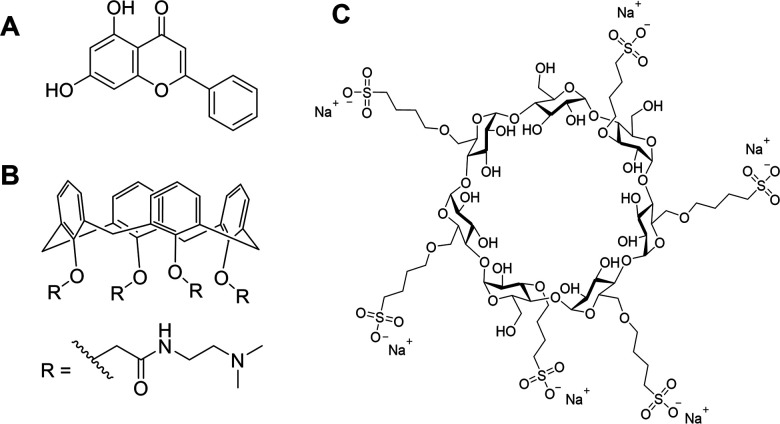
The chemical structure of (A) chrysin, (B) calixarene 0118 (OTX008) and (C) sulfobutylated β-CD (SBECD).^116^

As previously discussed, the ability to monitor glucose levels within the body is important for the management of diseases such as diabetes, known for causing conditions such as fibrosis. This condition occurs where extracellular deposits form on body tissues (*e.g.* heart, lungs, liver, and kidney) resulting in deleterious health effects.^115^ The drug chrysin (CHR), combined with calixarene 0118 (OTX008, Fig. 15A and B) are promising molecules for the treatment of fibrosis, however, the aqueous solubility of this system currently limits the use of these molecules to treat this condition.^116^ To remove this limitation, Fenyvesi and co-workers have incorporated a sulfobutylated β-CD (SBECD) into this formulation (Fig. 15C).^117^ Here it is hypothesised that the CHR forms host–guest inclusion complexes with both the SBECD and OTX008, while electrostatic interactions between the inclusion complexes create an aggregate structure that is able to entrap any unbound CHR.^117^ As a result, the formation of these supramolecular aggregates effectively removes the solubility limitations associated with CHR and OTX008, enabling progression towards application within the clinic for the treatment of fibrosis.

Cancer is one of the leading causes of death worldwide, responsible for 9.7 million fatalities in 2022,^118^ which is predicted to rise to 16.3 million deaths per year by 2040, this is due to factors such as drug resistance and the ageing global population.^119,120^ Therefore the global oncology market, which was valued at $242 billion in 2024, is also set to rise, with a predicted value of $522 billion by 2033.^121^ However, one of the greatest challenges associated with the development of novel anticancer strategies remains the selective targeting of cancer cells over normal cells, reducing undesirable side-effects.^122^

To overcome this limitation, Xu and co-workers have developed a glutathione (GSH) sensitive supramolecular polymer that enables the selective targeting of GSH expressing tumours with the anticancer drug doxorubicin (DOX).^123^ This supramolecular polymer is formed through the linking of multiple β-CD monomeric units using GSH sensitive *p*-nitrophenyl carbonate functionalities, that are in turn linked together through the use of disulfide bonds.^123^ Stability was further enhanced through the addition of hydrophilic amino poly(ethylene glycol) methyl ether (PEG-NH_2_) functionalities at the terminal ends of the β-CD incorporated polymeric chains, increasing the amphiphilic nature of the polymer. The DOX was able to form a host–guest inclusion complex with the β-CD, resulting in the formation of aggregated species, stable for at least seven days, with a hydrodynamic diameter of 109.96 nm ± 2.26 nm and a stability of −22 ± 0.8 mV, determined by dynamic light scattering/transmission electron microscopy and zeta potential measurements respectively.^123^ Glutathione is known to reduce disulfide bonds, so in the presence of cancer cells which express high levels of GSH, the disulfide bonds that are responsible for linking the monomeric units of this polymer together are broken, facilitating the release of the DOX cargo from the polymeric aggregate and enabling the selective targeting of these cancer cells. *In vivo* testing in mice confirmed this polymer to effectively reduce the size of tumours derived from 4T1 (human breast cancer) cells, while enabling the mice to maintain their weight over a 12 day period.^123^ In contrast to this, under analogous experimental conditions, where DOX was administered alone, although a reduction in tumour size was noted this was not as great as when the same drug was supplied when incorporated into the polymer and additionally, weight loss on the mice was observed. This observation was attributed to off-target cytotoxicity effects. Therefore, this supramolecular innovation demonstrates the potential for β-CD incorporated polymers to enable the targeted drug delivery of anticancer drugs, such as DOX, for the treatment of GSH producing cancers. These macrocyclic compounds have been shown to enhance anticancer drug efficacy and retaining system stability, while reducing cytotoxic effects against healthy cells *in vivo*.

**Fig. 16:**
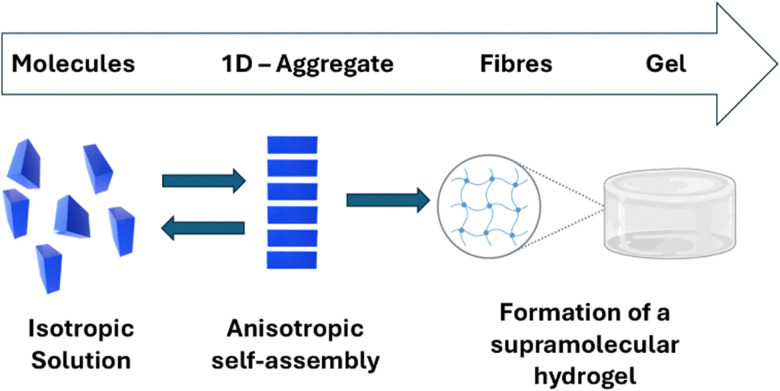
The hierarchical self-assembly of a hydrogel through the non-covalent interactions of monomers.^125^

Unlike conventional gels, which rely mainly on the formation of covalently cross-linked polymer networks, a supramolecular gel is produced through the self-assembly of small molecules (gelators) *via* the formation of non-covalent interactions (Fig. 16).^124,125^ This leads to the formation of solid fibres, which trap a liquid phase (the ‘sol’) to form the ‘gel’. Depending on the nature of the solvent that forms the bulk of this liquid phase, the gel can be further classified as either a hydrogel or an organogel.^124,125^ Supramolecular gels have demonstrated applications across various fields which include tissue repair, wound dressing, and drug delivery vehicles.^126,127^

**Fig. 17:**
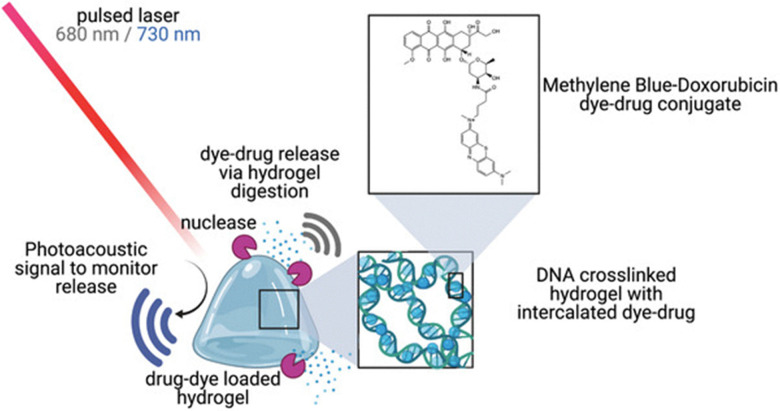
A biodegradable hydrogel that enables the monitoring of chemotherapeutic drug photoacoustically. Here, MB-Dox is loaded into a DNA cross-linked hydrogel. The MB-Dox retains an activatable wavelength-specific photoacoustic signal both when loaded and following drug release. Reproduced from ref. 129 with permission from Advanced Science copyright 2022.

In addition, supramolecular hydrogels have also been used as bioimaging agents, enabling the visualisation of organism structure, clarifying biological functions, and tracking biological processes (such as drug delivery) in real-time without physical interference.^128,129^ However, several additional capabilities are imperative for an image-based drug tracking system to become viable. These additional capabilities include the ability of a system to: (i) characterise the amount of a drug present and, (ii) achieve an activatable change in contrast.^130,131^ Although many supramolecular material bioimaging strategies exist, such as the use of flexible non-linear optical membranes, composed of CD[7] complexed with 4-*N*,*N*-dimethylamino-4′-*N*′-methyl-stilbazoliumtosylate that enable the real time imaging of *E. coli*,^132^ photoacoustic imaging (PAI) represents a leading method to image and monitor drugs *in vivo*.^133^ PAI uses near infrared (NIR) wavelengths to safely penetrate tissue and activate both nano- and molecular-scale contrast agents, while conventional ultrasound detects and reconstructs images based on the optical absorption of the tissue, a process summarised in Fig. 17.^134,135^

Although many systems have employed PAI to monitor *in vivo* drug delivery systems, few satisfy the aforementioned accessory capabilities that enable the development of a viable image-based drug tracking systems.^136,137^ However, in 2021 Jokerst and co-workers demonstrated that a supramolecular DNA hydrogel, originally developed by Wang and co-workers, loaded with methylene blue-doxorubicin (MB-Dox) conjugates, was capable of fulfilling these accessory capabilities enabling real-time PAI of drug release (Fig. 17).^129^ Here the MB-Dox is incorporated into the DNA hydrogel through intercalation into the DNA double helix contained within the hydrogel fibres. This process resulted in a 91.3% drug loading efficiency.^129,138^ The cytotoxicity of MB-Dox was subsequently established against SKOV3 ovarian cancer cells. Further studies confirmed the MB-Dox incorporated DNA hydrogel system enabled prolonged and localised drug release, while simultaneously maintaining a strong photoacoustic signal (to enable PAI) the intensity of which correlated with drug release and biodistribution.^129^

Finally, the antitumour efficacy of the MB-Dox incorporated DNA hydrogel was established in mice.^129,139^ Mouse tumour burdens were monitored by observing the alterations in bioluminescent signals over 14 days. After the first week, mice that had been subcutaneously injected with the MB-DOX loaded DNA hydrogel showed a significant decrease in tumour bioluminescence. After two weeks, treatment with the hydrogel resulted in a 77.91% reduction in tumour burden.^129^ Not only do these results highlight the therapeutic capabilities of the MB-Dox incorporated DNA hydrogel, but also the utility MB-Dox as a non-invasive, real-time system for monitoring chemotherapeutic efficacy.^129^

Supramolecular gels have also been developed to prevent the deterioration of medical implants. Traditionally, hydroxyapatite (HA), a primary component in bones and teeth, has been applied to the surface of metallic implants to enhance biocompatibility.^140,141^ However, the presence of this coating also accelerates implant deterioration, which occurs as the result of increased friction and leads to an increased susceptibility towards bacterial infection.^141,142^ Ha and co-workers have innovated within this area, removing these limitations.

**Fig. 18:**
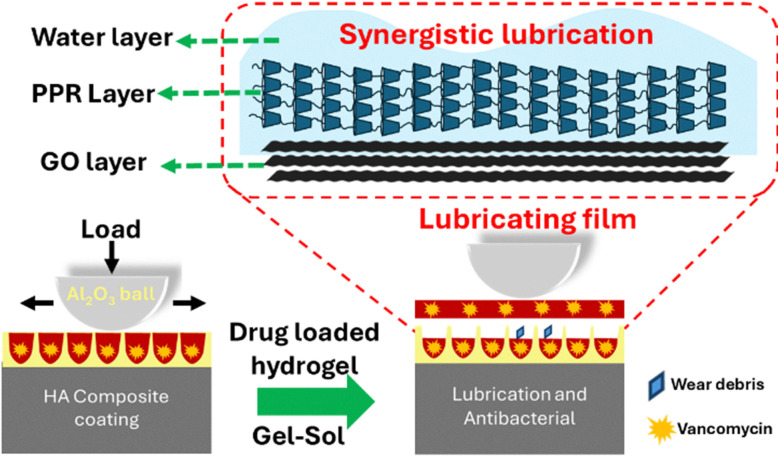
The structure of hydroxyapatite (HA) hydrogel structure and gel–sol transitions under a load.^143^

Here craters were incorporated within the HA surface, before the surface was coated with a hydrogel.^143^ This hydrogel was produced *in situ via* host–guest complexation between amino poly(ethylene glycol) methyl ether (GO/PEG-NH_2_) and α-CD in the presence of graphene oxide sheets.^143^ The hydrogel was subsequently infused with the antibiotic vancomycin, to reduce the infection rates presented with previous generation of HA coated metallic implants, Fig. 18.^143–145^

As force was applied at the surface of the hydrogel, the material undergoes a gel–sol transition, with the resultant sol mimicking articular synovial fluid, while the craters within the HA surface collect any debris. Tribology studies showed the hydrogel coating reduced the friction coefficient by five times (0.43 to 0.089) and wear rate by three orders of magnitude (1.39 × 10^−2^ mm^3^ N^−1^ m^−1^ to 1.07 × 10^−5^ mm^3^ N^−1^ m^−1^) *vs.* the HA surface alone.^143^

The antimicrobial efficacy of the vancomycin loaded hydrogel was confirmed against clinically relevant *Staphylococcus aureus*. Appreciable antimicrobial efficacy was demonstrated at a drug loading of 80 μg mL^−1^ of hydrogel.^143^ The vancomycin release properties of this hydrogel confirmed a fast release of the antibiotic over the first 12 hours, which subsequently slowed. After 10 days, 89% of the vancomycin had been released from the hydrogel and after 15 days, 94% of the drug was released.^143^ It was hypothesised that the increased electrostatic interaction between the positively changed vancomycin and negatively charged graphene oxide sheets incorporated into the hydrogel supported this long-term drug release.^143^

##### Commercial case study: Innovotex

The discovery and application of Texaphyrins in oncology by Sessler and co-workers has capitalised on the ability of texaphyrins to amass within cancerous tissue, and lead to the formation of Pharmacyclics Inc. in 1991.^146–148^ The subsequent development of ibrutinib, a Burkett tyrosine kinase (BTK) inhibitor, lead to the sale of Pharmacyclics Inc. to AbbVie, a global biopharmaceutical company, in 2015 for $21 billion.^149^

**Fig. 19:**
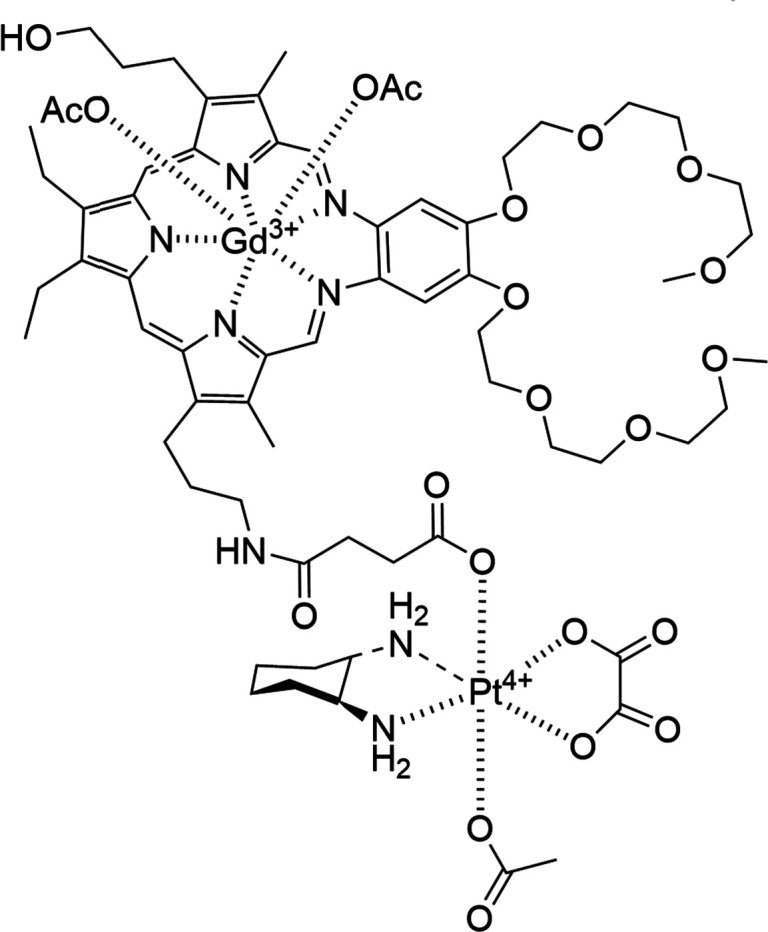
The structure of the texaphyrin based oxaliplatin drug conjugate OxaliTex.^150^

Following on from this success, Sessler and co-workers developed texaphyrins as cancer drug carriers for platinum-based drugs (*e.g.* cisplatin, carboplatin, and oxaliplatin), principally for use against ovarian cancer. Development in this area lead to the design of OxaliTEX, a texaphyrin based oxaliplatin drug conjugate (Fig. 19).^150^ Here the inclusion of the diaminocyclohexane ligand produced a molecule that could overcome the resistance mechanisms of platin-resistant wild type p53 ovarian cancer cells *via* ribosomal stress induction. This lead compound also displayed activity comparable to carboplatin against A549 lung cancer and A2780 ovarian cancer cell lines.^150–152^

OxaliTex contains a platinum centre capable of forming both six coordinate and four coordinate complexes in tetravalent and divalent states respectively. The axial ligands have two functionalities, the first is to bind the oxaliplatin to the MGd texaphyrin core and the second is to bind to the acetate anion, providing a balance between kinetic stability, while also acting as a good leaving group under reduction conditions such as certain tumour microenvironments.^147^ When reduction occurs, bonds to the oxaliplatin also cleave resulting in effective drug release.

*In vivo* mice model studies have verified the maximum tolerated dose of OxaliTEX to be around three times higher than that of oxaliplatin. A xenograft model derived from platin-resistant wild type p53 ovarian cancer cells showed that neither the vehicle control nor oxaliplatin had any effect on tumour growth however, OxaliTEX was shown to successfully prevent any further tumour growth over the course of 14 days.^150^

OxaliTEX has demonstrated vast potential as a targeted anticancer therapeutic, demonstrating a high degree of toxicity toward cancerous cells and limited cytotoxicity towards normal cells. This selectivity is attributed to the tumour localising characteristics of texaphyrins. Innovotex is actively working toward the clinical development of OxaliTEX as a viable drug for the treatment of platinum drug resistant ovarian cancers. The steps towards this are in progress, with first patient studies planned for 2026.^153^

### Applied supramolecular materials

#### Self-healing polymers, coatings, and rubbers

Many conventional polymers – including those commonly found in the household, like polyethylene terephthalate, polyvinyl chloride, polyethylenes, and polypropylene – are produced through the formation of covalent bonds which form between the monomeric units,^154^ producing ‘spaghetti-like’ structures that entangle.^155^ This leads to the production of materials that are difficult to recycle due to high melt viscosities and that may respond undesirably to environmental factors.^156^ Supramolecular polymers are assembled through reversible, highly directional non-covalent interactions along their main chain, which hold the structure together.^157^ This reversibility causes this class of polymer to form under thermodynamic equilibrium, with chain length determined by factors including the strength of interactions, monomer concentration and temperature. Unlike conventional polymers, the dynamic nature of these non-covalent bonds imparts unique and responsive properties to supramolecular systems, while simultaneously reducing melt viscosities.^158–160^

Stimuli-responsive supramolecular polymers can be designed to react to a variety of different environmental factors, including but not limited to temperature,^161^ stress,^162–164^ pH,^165–167^ and the presence of other molecular species.^168,169^ These responses are enabled by non-covalent interactions which can grant the materials unique capabilities such as enhanced recyclability,^170^ self-adaptability,^171^ or water retention,^172^ making them useful in a range of commercial applications.

**Fig. 20:**
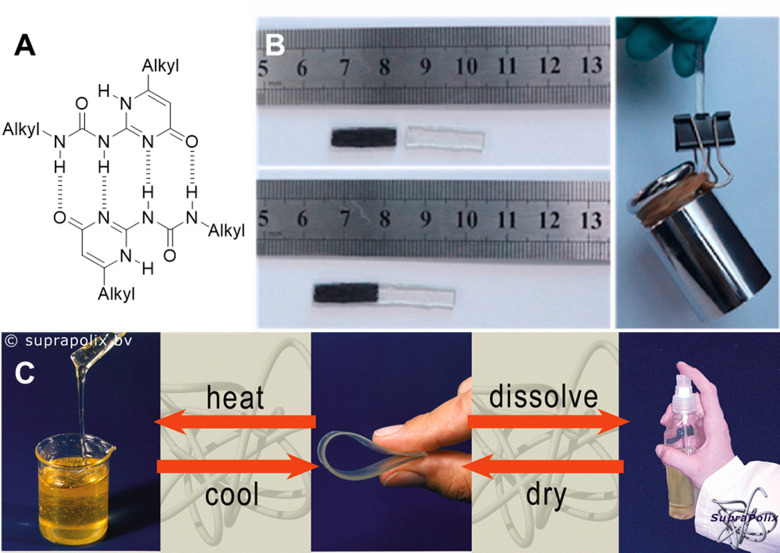
(A) The dimer structure of UPy, highlighting the 4 hydrogen bonds formed. (B) The self-healing and the strength capable of the UPy containing polymer from reproduced from Zhang *et al.*^180^ with permissions from Elsevier, copywrite 2022. (C) The phase changes of Suprapolix produced, UPy modified Kraton®, (poly(ethylene-*co*-butylene). Highlighting the phase behaviour change due to the supramolecular interactions. Reproduced from Bosman *et al.*^181^ with permission from Elsevier, copyright 2004.

Self-healing polymers, coatings, and rubbers represent specific sub-categories of this class of supramolecular material, that when damaged can undergo self-repair, without any outside intervention.^173^ This property can yield materials that are ‘maintenance free’, prolonging the lifetime of products that they are incorporated into.^174^ Meijer and coworkers have incorporated UPy as a key motif in supramolecular polymers. UPy forms strong yet reversible dimers through the multiple cooperative hydrogen bonds (*K*_dim_ = 6 × 10^7^ M^−1^ in CHCl_3_ at room temperature),^175–177^ making it an ideal candidate for supramolecular polymerisation, as shown in Fig. 20A. Meijer and co-workers have taken advantage of this, incorporating UPy into reversible networks,^178,179^ however in recent years UPy has also been explored for its application in self-healing materials. Polyurethane is a widely used commercial material that is prone to cracking and fatigue over time. Zhang *et al.*^180^ have integrated UPy into a polyurethane elastomer to remove this limitation. This UPy functionalised polyurethane elastomer recovers to 87.7% of the original tensile strength at room temperature after being fractured. In addition, this functionalisation also enhances fluidity and simple physical blending to ease the manufacturing process (Fig. 20B).

##### Commercial case study: Suprapolix

Quadruply hydrogen-bonding UPy underpins several different technologies marketed by the company Suprapolix.^181^ This company, founded in 2002, has effectively expanded the SupraB^TM^ portfolio. Within the SupraB^TM^ portfolio is a self-healing elastomer capable of self-adhesion (Fig. 20C); a self-healing coating which heals cuts and scratches when heated; and a hydrogel that flows like a liquid when heated but acts like a rubber again when cooled. Their materials aim to provide unique processing and rheological benefits with tuneable material performance, with the underlying principles patented for use within self-healing polymers,^182–185^ coatings,^186^ hydrogels,^187,188^ and biomedical applications.^189^

**Fig. 21:**
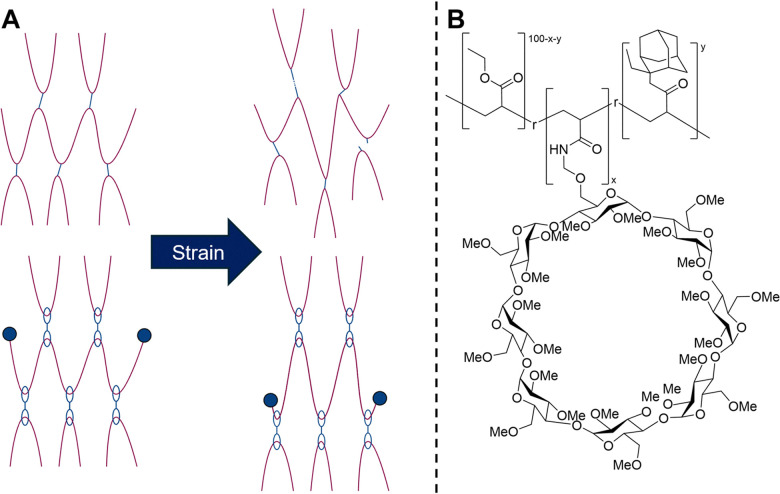
(A) Schematic overview of crosslinked polymers *versus* slide-ring polymers and their differing response to strain. (B) The structure of the supramolecular elastomer, PAcγCDAAmMe produced by Nomimura *et al.*^196^

In our previous review, we reviewed work by Harada and co-workers in utilising the mechanical bond in slide-ring polymer systems (Fig. 21A).^190,191^ Since this time, Takashima and co-workers have built upon these concepts to utilise host–guest complexation to provide self-healing in a range of patented polymeric systems.^192–195^ In 2019, their team synthesised self-healing acrylate-based elastomers with hosts – functionalised CDs PMγCDAAmMe, PAcβCDAAmMe, and PAcγCDAAmMe – and guests – ethyladamantyl acrylate (AdEtA) and fluorooctyl acrylate (H2F6, Fig. 21B).^196^ When damaged, the material was able to return to 99% of the initial strength within four hours, a phenomenon attributed to the host–guest cross-linking interactions. These materials were also shown to be highly flexible and tough elastomers, with fracture energies up to 800% and strengths 12-times greater than conventional cross-linked polymers.

In 2020 Sinawang *et al.*^197^ combined a host monomer, the acyl functionalised cyclodextrin, PAcγCD; a guest monomer, 12-acrylamido dodecanoic acid; a main chain monomer, 2-hydroxyethyl acrylate; and citric acid-modified cellulose through radical copolymerisation to form a hydrogen bond reinforced, slide ring polymer. This self-healing material shows improved strength compared to previous self-healing materials, with strengths able to reach a strain of 1000%. Self-healing ratios of 56% at room temperature and 84% at 80 °C were achieved, whilst also giving the additional benefits such as high tensile stress (21 MPa) and high fracture energy (151 MJ m^−3^) which are comparable to low-density polyethylene (LDPE). This was achieved through the dual non-covalent interactions of the host–guest system and the hydrogen bonding between the alkyl derivatives and the carboxyl and hydroxyl groups of the citric acid-modified cellulose.

**Fig. 22:**
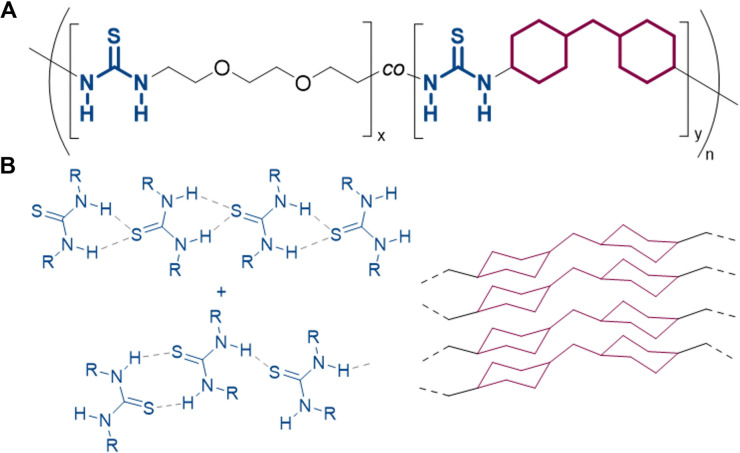
(A) The structure of P(TUEG_3_-*co*-TUCy_2_M). (B) The hydrogen bonding patterns of thiourea and the tacking patterns of dicyclohexylmethane units.^198^

Related work in this area by Aida and co-workers has shown that poly(ether thiourea) (PTUEG_3_) containing polymers self-healed at ambient temperatures.^198^ However, under high humidity, PTUEG_3_ absorbed water and was plasticised, losing its mechanical strength. Aida and co-workers published works in 2021, overcoming the issues encountered when under high humidity.^199^ A copolymer containing the monomer unit of (PTUEG_3_) and 10% dicyclohexylmethane thiourea (TUCy_2_M) was devised to serve as a humidity-tolerant, mechanically robust polymer that can self-heal at ambient temperatures (Fig. 22). The dicyclohexylmethane units stack tightly when they bare adjacent hydrogen bonding units,^200–203^ allowing for high humidity tolerant, self-healable polymer. The patent relating to this technology was published in 2021.^204^

More recently, Aida and co-workers also developed poly(thioether thiourea), a glassy polymer capable of self-healing at temperatures well below their glass transition temperature.^205^ Here the introduction of a disulfide bond enhanced the healing properties of the system by taking advantage of dynamic covalent nature of the metathesis-active disulfide bonds. Furthermore, poly(thioether thiourea)s are much less hygroscopic than PTUEG_3_, making the resultant materials less prone to plasticisation.

**Fig. 23:**
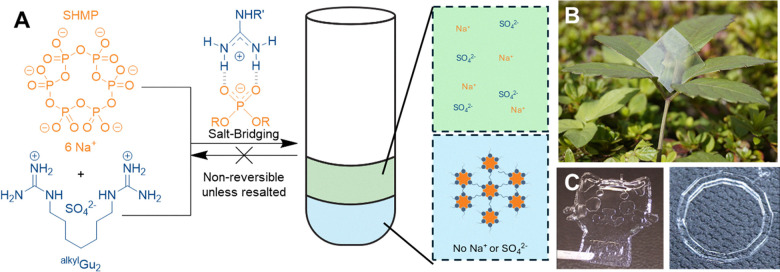
(A) Molecular structures of SHMP (orange) and a Gu-based monomer (^alkyl^Gu_2_) (blue), spontaneously liquid–liquid phase separating upon mixing at a molar ratio of 1 : 3. Leading to two macroscopically separated phases: a water-rich upper phase containing Na^+^ and SO_4_^2−^, and a condensed lower-phase containing a 3D cross-linked supramolecular polymer network between HMP and ^alkyl^Gu_2_. (B) A plastic film formed by the evaporation of the condensed phase followed by hot pressing. Made available by the Aida lab at https://www.aidacreativehub.com/. (C) 3D printed objects from the Gu-ChS polymers. Reproduced from work by Cheng *et al.*^206^ under creative commons license CC BY 4.0.

To achieve more environmentally benign materials, the same group also developed closed-loop recyclable, supramolecular plastic.^206,207^ When the ionic monomers sodium hexametaphosphate (SHMP) and di- and tri-topic guanidinium (Gu) ions are combined in aqueous conditions a multivalent cross-linked structure forms spontaneously through liquid–liquid phase separation, Fig. 23. The hydrogen bond reinforced, salt bridges mean that the plastics themselves are very strong, thermally reshapable, but metabolizable. After being resalted, the salt-bridges are disrupted, allowing SHMP and Gu to be metabolised by microorganisms when in biologically relevant conditions, *e.g.* sea water. Gu was also combined with the biosourced polysaccharide, chondroitin sulfate (ChS). This formed similar salt bridges and yielded a supramolecular polymer suitable for 3D printing.

#### Adhesives

During the manufacturing process, materials are often joined using adhesives such as epoxy and urethane, which rely on chemical affinity between the adhesive and the adherend.^208^ The strength of the bond is determined by the chemical or physical compatibility between the adhesive and the surfaces being joined.^209^ Once cured, the hardened adhesive becomes embedded in the surface of the materials to be joined, creating a strong bond through an anchoring effect. Although these adhesives provide high strength, they often suffer from limited toughness, poor stress resistance, and low stretchability.^210^ In cases where chemical bonding is absent, adhesion primarily depends on van der Waals forces.^211^ While these interactions are relatively weak compared to other interactions, they can enable reversible adhesion.^212^

Supramolecular adhesives are seeking to address these challenges, overcoming low stress resistance of chemically bonded adhesives and the weak adhesion associated with van der Waals interactions. These next-generation adhesives leverage supramolecular interactions to enhance interfacial bond strength while also offering improved recyclability, greater energy dissipation, and faster bonding.^213^

**Fig. 24:**
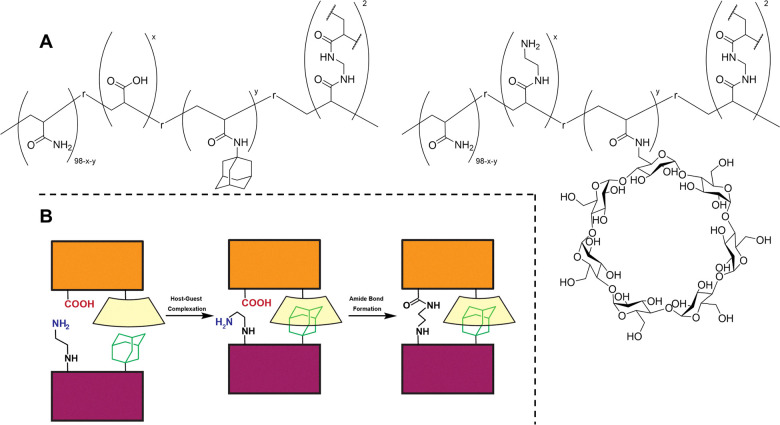
(A) The structures of the two hydrogels formed by Osaki *et al.*,^215^ containing the host–guest units of β-CD and Ad and the condensation units of carboxyl and amino groups. (B) Schematic overview of the host–guest interaction facilitating the correct spatial distance to facilitate the condensation reaction.

Takashima and co-workers have explored and patented work utilising host–guest interactions to provide favourable and novel characteristics to adhesion systems.^192–195,214^ In 2021 Osaki and co-workers^215^ used host–guest interactions between (β-CD) and adamantyl (Ad) groups to assist the condensation of amino groups with carboxyl groups, a common motif in adhesive technologies, Fig. 24. The host–guest interaction provides the appropriate spatial distance for the functional groups to form the amide bond, whilst also providing a greater adhesion in the presence of a condensation reagent, when compared to van der Waals forces. The number of host–guest units showed good correlation with adhesion strength and increased the rupture strength of the material after being treated with condensation reagents. It was also seen in every tested host–guest ratio that the rupture strength increased after treatment with condensation reagents.

**Fig. 25:**
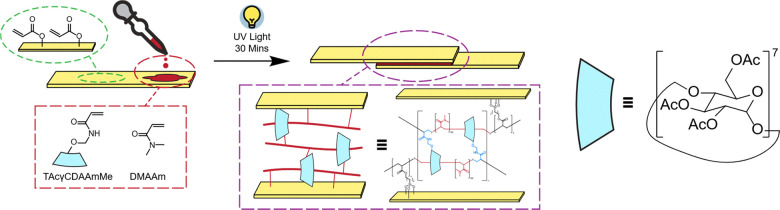
Schematic of the fabrication and structure of SC(DMAAm).^216^

Qian *et al.*^216^ have sought to add energy dissipation to adhesive materials by employing the slide ring polymer strategy (Fig. 21a). A mixture of copolymers *N*,*N*-dimethylacrylamide (DMAAm) and triacetylated 6-arylamido methyl ether-γ-CD (TAcγCDAAmMe) with photoinitiator IRGRACURE was applied between two acryl-functionalised Nylon-66 substrates (Fig. 25). The system was then irradiated with UV light to induce dynamic, slide-ring cross-threading. The supramolecular adhesive SC(DMAAm) formed as a result demonstrated a greater adhesion strength, toughness, energy dissipation, self-restoration, and creep properties when compared to the control cross-linked and linear polymer systems.

Movable cross-links facilitated by mechanical bonds were also utilised in a collaboration between Takashima, Arai, and co-workers to provide tough, soluble, recyclable adhesive sheets.^217^ Here, a recyclable slide-ring adhesive was prepared by dissolving triacetylated 6-arylamido methyl ether-β-CD (TAcβCDAAmMe) and radical initiator OMNIRAD 184 in ethyl acrylate and injected into a reaction cell. The cell was irradiated with UV light for bulk polymerisation to provide the elastomer sheet, M-PEA-CD. M-PEA-CD provided higher peel strength and creep resistance when compared to the linear, poly(ethyl acrylate) and A-DCP containing cross-linked polymer, CC-PEA. As it was also soluble in organic solvents, M-PEA-CD was able to be solvated and remoulded through evaporation of the solvent to give a recycled M-PEA-CD sheet. The adhesive properties of the recycled sheet were shown to be retained.

A similar strategy was employed by Matsumura *et al.*^218^ to form stimuli-responsive degradable adhesive materials. TAcγCDAAmMe, the photoinitiator OMNIRAD 184, and the photoacid generator bis(cyclohexyl sulfonyl) diazomethane (BCSD) were dissolved in ethyl acrylate. 365 nm light was irradiated at the solution for 120 minutes to form the elastomer PEA-TAcγCD/BCSD. When irradiated with 254 nm light, BCSD undergoes denitrification, and the degraded product reacts with H_2_O to forming sulfonic acid. The strong Brønsted acid cleaves the *o*-amidomethyl bond through a hydrolysis reaction and releases the threaded polymer chains. This caused the adhesion strengths to drop by 37%, 25%, and 53% on Nylon 66, SU303, and glass substrates respectively.

In 2024, Aida and co-workers published their serendipitous discovery using poly(ether thiourea) as an all-underwater adhesive,^219^ after being patented in 2023.^220^ Despite being more acidic than urea, thiourea exchanges its N–H protons with water 160 times slower at 70 °C, suggesting that thiourea is much less hydrated than urea in an aqueous environment. From their work in 2018,^198^ it was noticed that the polymer strongly adhered to wet glass, leading to the principle that thiourea functions as a ‘polar hydrophobic’ hydrogen bonding motif, due to the irregular shaped hydrogen bonding network which discourages hydration layers within the network. When compounded with the inclusion of a dicyclohexylmethane monomer unit to minimise the negative influence of water through non-covalent stacking, an adhesive capable of all-underwater adhesion that lasts for a full year can be made. The all-underwater adhesion worked on multiple substrates and was able to support the weight of 60 kg when adhered on to two steel plates with an adhesion surface area of only 8 cm^2^.

#### Gas storage

Gas storage technologies have attracted significant attention in recent decades due to global decarbonization efforts and the need to replace energy-intensive separation methods like cryogenic distillation.^221^ Innovations in hydrogen storage are highly sought after to realise the potential for hydrogen to replace fossil fuels as a greener energy source.^222^ Several types of porous materials, such as covalent organic frameworks, zeolites, activated carbon nanotubes (CNTs) or metal organic frameworks (MOFs),^223,224^ have been identified, scaled up and commercialised for gas uptake and storage applications. Supramolecular approaches have typically centred around constructing porous materials (solids and liquids) with intrinsic pores; these are predominantly synthesised as solids under ambient conditions, restricting their use in water-based applications and necessitating crystalline structures for accurate characterization. Many also face oxidative instability and weak selectivity, particularly against water vapor in humid environments. Recyclability remains challenging for some systems, while others show performance that varies significantly with target gas concentrations. A critical challenge lies in designing materials capable of concurrently overcoming these interrelated limitations.

Since 2021, progress has been made in exploring alternative synthetic routes towards porous organic cages (POCs), which could ultimately improve their scale-up potential for industrial use.^225–228^ Additionally, the increased availability of laboratory robotics has opened up new opportunities for automated screening and may facilitate more informed rational design and automated production in future.^229,230^ There have been notable advances in computational analysis of porous solids, offering insight into the stability and shape persistence of cages and how they assemble in the solid state (*via* crystal structure prediction approaches), all of which can advance understanding of solid, porous materials at a molecular level.^231,232^ The expanding toolkit of POC synthesis and analysis methods, including novel approaches beyond traditional non-covalent strategies, provides a critical pathway to scale these materials from lab research to practical applications.

##### Commercial case study: Porous Liquid Technologies

**Fig. 26:**
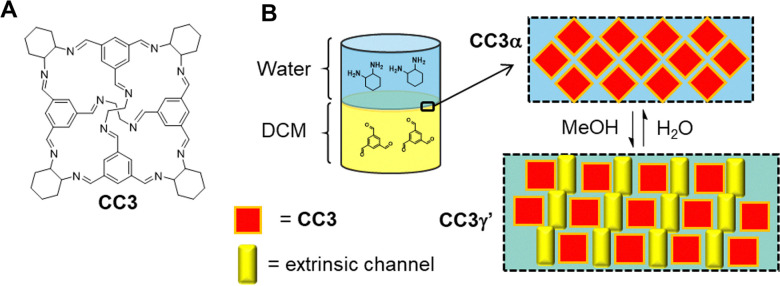
(A) CC3, a porous organic cage developed by Cooper and colleagues;^240^ (B) the formation of crystalline CC3 membranes at the interface between two immiscible solvents. The membranes reversibly transition between two crystalline phases in response to the solvent environment.^244^

Cooper and James have been pioneers in the field of porous organic cages (POCs) for over a decade, focusing on their incorporation into both porous solids and porous liquids.^233–235^ Their research has led to the development of various POC systems capable of CO_2_ absorption,^236^ hydrogen isotope separation,^237^ SO_2_ capture,^238^ and noble gas capture.^239^ A patent describing the stabilisation of porous materials based on Cooper's core cage architecture (Fig. 26A) was granted in 2023,^240^ following the launch of UK spin-out Porous Liquids Technologies in 2017.^241^

For crystalline POCs to find application in filters and other devices, a route to immobilising them within robust, solid materials is needed to avoid the drawbacks of handling fine powders. It is crucial that the adsorption capabilities of the POC are not compromised by the immobilisation process;^243^ developing such post-synthetic processes has been an area of recent progress in the field. In 2022, Livingston and Cooper reported a crystalline porous organic cage that could be fabricated into a membrane with switchable apertures for molecular sieving applications.^244^ The membranes were grown at the interface between two immiscible solvents; a dichloromethane layer containing 1,3,5-triformylbenzene and a water layer containing (1*R*,2*R*)-1,2-diaminecyclohexane (Fig. 26B). This led to the formation of free-standing films of a porous organic cage, CC3, which could be transferred onto various substrates producing a highly crystalline and defect-free film with a constant and continuous thickness of 80 nm.

The crystallinity and crystal phase (CC3a) of the membranes produced was confirmed by powder X-ray diffraction (PXRD) and grazing incidence X-ray diffraction (GIXRD) measurements. The authors found that submersion in methanol transformed the film into a new, crystalline structure which was found to be a distinct, methanol-solvated crystal phase (CC3g′), which is very different from the starting phase. In particular, the density of cages within the CC3a phase is much higher with window-to-window packing, while the cages in CC3g′ are less densely packed with large extrinsic pores between cages. These differences in crystal packing and hence porosity were exploited for switchable molecular sieving applications; the molecular weight cut-off shifted from 600 g mol^−1^ in water to 1400 g mol^−1^ in MeOH for the same membrane. To demonstrate this principle, the authors showed that the rejection of Brilliant Blue dye (BB, MW = 826 g mol^−1^) was 100% in water and 0% in methanol. They also performed a graded sieving experiment in which a mixture of 4-nitrophenol (NP; MW = 139 g mol^−1^), BB and Direct Red 80 (DR; MW = 1373 g mol^−1^) in water was filtered through the CC3 membrane. Initially only NP could pass through the membrane in the CC3a phase; flushing with methanol then transformed the membrane to the CC3g′ phase, enabling BB to pass through and leaving only DR in the retentate.

In 2024, García-Tuñón and colleagues reported that hybrid porous materials containing CC3 could be 3D-printed using a direct ink writing (DIW) approach.^242^ DIW involves the extrusion of an ink material from a nozzle to form a continuous, self-standing material.^245^ A key challenge was to initially formulate CC3 into a paste with the requisite rheological properties for DIW, but without the use of additives which could block the pores of the cages and render them inaccessible in the final material. Here, the authors formulated crystalline CC3 powder with graphene oxide (which is known to facilitate non-covalently bridged cage networks), 6 wt% F127 hydrogels and a bentonite clay matrix in water, which yielded an ink with optimal flowability for printing and minimal crack formation upon drying. Crucially, the adsorption properties of the CC3 were retained; the 3D printed material containing 20 wt% CC3 had a loss of ∼25% capacity compared to the theoretical value.

In addition to optimising the ink formulation, 3D-printing is a versatile method which allows fine-control over the geometric features of the materials being produced; these can be optimised to benefit different applications. Here the authors explored several grid-type structures with differing internal geometries, and explored the effect on the N_2_ sorption properties of the material. This experimental work was combined with computational flow dynamics simulations to understand how the 3D-printed geometry affected the circulation of gases through the material. This combined experimental and computational approach has great potential to optimise function on a molecular and macroscopic level.

In this same year, Stoddart and co-workers reported a supramolecular approach to enhancing the volumetric hydrogen storage capacity in supramolecular crystals.^246^ Materials comprised of lightweight, organic molecules such as hydrogen-bonded organic frameworks (HOFs) have a high gravimetric surface area due to their low weight. However, the volumetric capacity is arguably more important given considerations such as the low volume of storage tanks in automobiles, and many materials with high gravimetric capacity have poor volumetric capacity. Interpenetration can also reduce the volumetric surface area of a material.

**Fig. 27:**
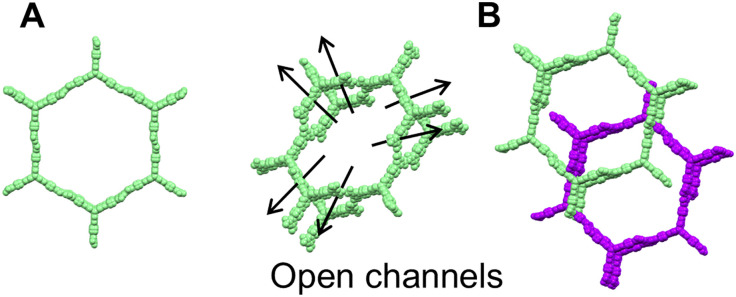
(A) Two views of the hexagonal units within the 3D honeycomb-like frameworks in the HOF reported by Stoddart and colleagues for hydrogen storage; (B) catenation of two hexagons linking two distinct layers (green and purple) within the HOF material.^246^

In this work, the authors hypothesised that catenating a HOF material could prevent interpenetration and avoid the loss of accessible surface area, whilst providing additional stability to the structure (Fig. 27). The catenated structure required careful geometric design to minimise the overlap area between interlocked components and thus yield a material with enhanced rather than reduced internal surface area. Based on these considerations the authors designed a rigid triptycene molecular skeleton incorporating carboxylates at each terminus for cross-linking the HOF material and an internal imidazole ring to provide a directing hydrogen bonding interaction for catenation. 3D honeycomb-like frameworks were obtained when these building blocks were crystallised from a DMF solution. Each 2D layer was comprised of arrays of hexagonal motifs, containing open channels through which inter-layer catenation could occur, driven by the formation of [N–H⋯O] and [O–H⋯N] hydrogen bonds. This yielded highly catenated structures which were stable up to 375 °C with good retention of crystallinity, which is comparable with robust MOFs.^247^ Importantly, the authors found that one of their materials exhibited desirable gravimetric and volumetric hydrogen-storage capacities (9.3 ± 0.2 wt% and 53.7 ± 1.0 g L^−1^ respectively), both exceeding the US Department of Energy targets of 6.5 wt% and 50 g L^−1^ for application in fuel cell vehicles.^248^

#### Supramolecular sensors

**Fig. 28:**
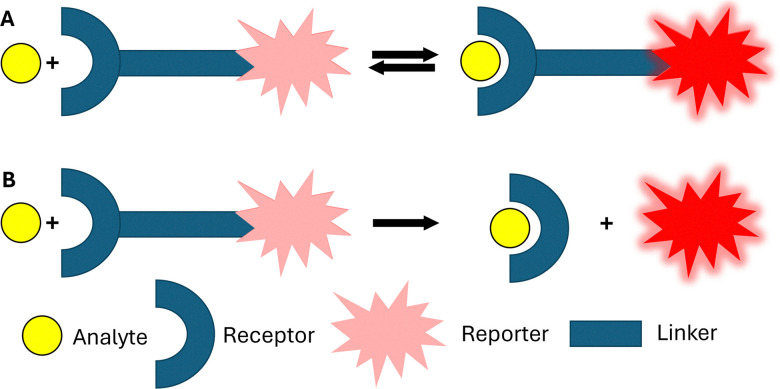
(A) Reversible binding of an analyte to a chemosensor. (B) Irreversible reaction-based recognition of an analyte with a chemodosimeter.

The detection and quantification of analytes is key for a range of biomedical and environmental technologies and is one for which the application of supramolecular chemistry is particularly well suited. Such sensors generally consist of a receptor, a unit which interacts or reacts with an analyte (in the case of a chemosensor or chemodosimeter respectively), and a reporter, which produces a measurable change in response to this interaction/reaction, Fig. 28. The choice of reporter is governed by the final application, but are commonly fluorescent, colorimetric or electrochemical outputs.

##### Commercial case study: SmartWound®

**Fig. 29:**
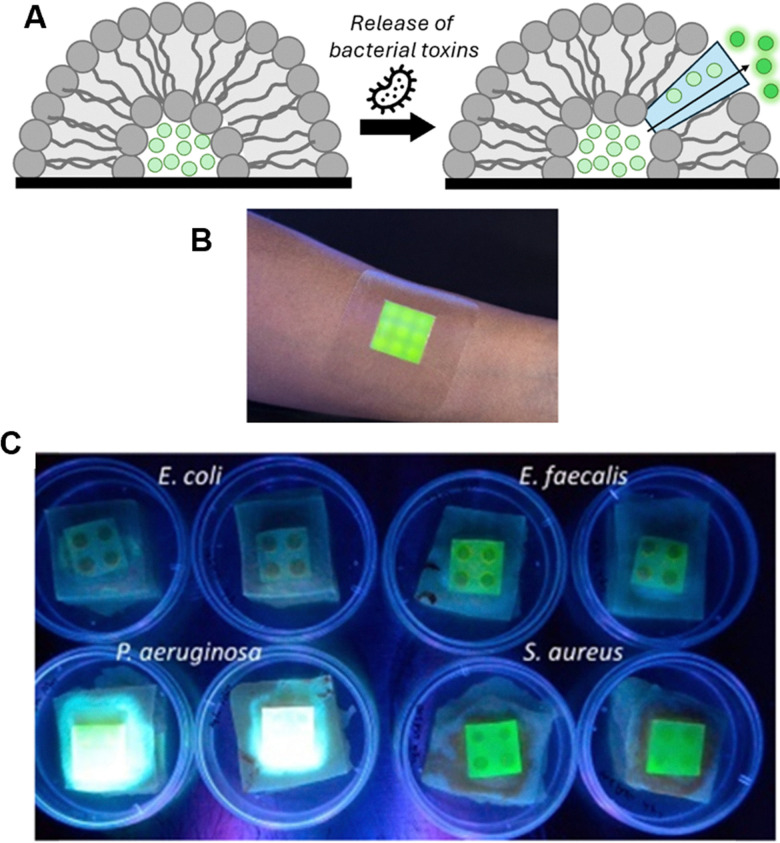
(A) A general schematic showing the insertion of a pore-forming toxin released by bacteria into the phospholipid vesicle membrane, triggering the release and ‘turn-on’ of a self-quenched fluorescent dye. (B) A photograph of the working wound dressing under visible light. (C) A photograph of the wound dressing model demonstrating its specificity of *Enterococcus faecalis*, *Pseudomonas aeruginosa* and *Staphylococcus aureus* over *Escherichia coli*. (B) and (C) Reproduced with from ref. 251 and 252 with permission from ACS, copyright 2016.

Due to their complexity, direct detection and differentiation of live cells is problematic. Jenkins and co-workers circumvented this problem by focussing on toxins specifically excreted by various bacterial pathogens, to develop a sensing technology capable of early infection detection in a clinical setting. This system is based around the self-assembly of phospholipid vesicles encapsulating the self-quenching fluorescent dye fluorescein.^249^ Pathogenic microbes including *Staphylococcus aureus*, *Pseudomonas aeruginosa*, *Candida* sp. and *Enterococcus faecalis*, produce proteinaceous pore forming virulence factors at bacterial concentrations related to the critical colonisation threshold, a bacterial concentration beyond which infection is likely to occur.^250^ These pore forming toxins permeabilise the vesicle membrane, releasing the self-quenched dye causing a concomitant turn on of fluorescence and a colour change (Fig. 29). The group have applied this technology to infection detection in burn wound infection both as suspensions within hydrogel wound dressings and as a swab for point of care testing in low resource settings.^251,252^ By altering the composition of the vesicle membrane, the group have also shown that microbe specificity can be tuned, developing a rapid point of care test for *Streptococcus agalactiae* (group B *Streptococcus*), a common cause of neonatal disease.^253,254^ This technology underpins the company “SmartWound®”, which spun out in 2021 and seeks to commercialise these diagnostic sensors.^255^

**Fig. 30:**
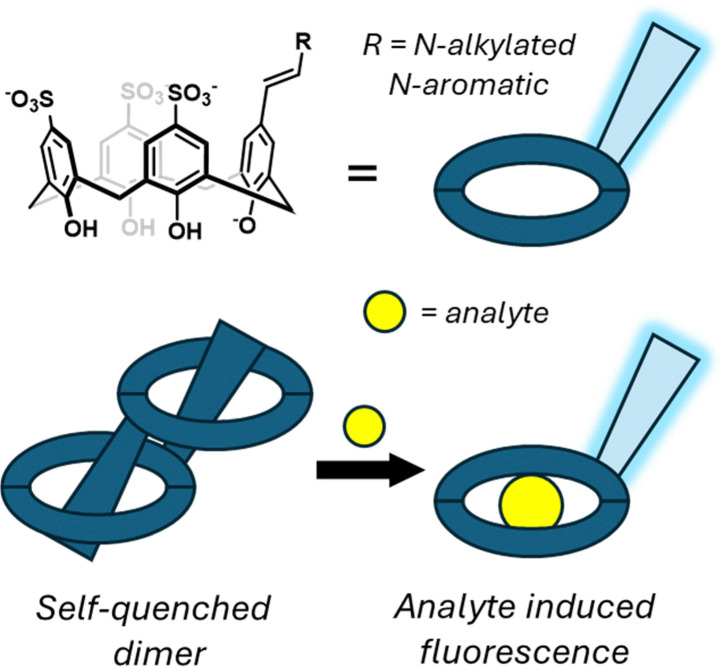
A schematic representation of the self-quenching an analyte induced fluorescence turn on mechanism of the dimer dyes.^257,258^

One of the key challenges in the design of supramolecular sensors is the design of receptors that are selective for a specific for specific guest species. Failure to achieve this makes the analysis of mixtures difficult, since whilst a sensor may interact more strongly with the analyte of choice, background activity of other similar species may present false positive readings.^256^ One method to circumvent these issues is to use multiple complimentary sensors in an array, in combination with statistical techniques, such as principal component analysis or linear discriminant analysis, to categorise and ‘fingerprint’ samples.^256^ The Hof group have developed the ‘DimerDyes’, a series of merocyanine appended calixarene macrocycles which dimerise and self-quench.^257,258^ These self-association events differ slightly with each different DimerDyes, owing to the difference in the merocyanine component. On exposure to various analytes in biologically relevant media (*i.e.* saliva), different analyte specific fluorescence responses could be measured from each of the different DimerDyes, as the analyte competes with the homo-dimerisation process (Fig. 30). Both principal component analysis and linear discriminant analysis were shown to be capable of classifying different opioids (heroin, 6-monoacetylmorphine, oxycodone, oxymorphone, dextrorphan), amphetamines (methamphetamine, amphetamine, methylenedioxymethamphetamine (MDMA), methylenedioxyamphetamine), and anaesthetics (cocaine, benzoylecgonine, lidocaine, procaine).^258,259^ This approach takes advantage of small differences in supramolecular interactions to develop a powerful tool to detect and discriminate illicit drugs.

Hof and co-workers have also developed an alternative to this classical multi-sensor approach, in which sensors are generally separated (*i.e.* in wells of a plate) using a one-pot adaptive supramolecular network.^260^ This concept relies on equilibria between not only sensors and analytes, but also the sensors with other sensors. This sensing system utilised the groups previously published ‘DimerDyes’; by using a mixture of three DimerDyes with distinct photophysical characteristics, a dynamic system is formed that consists of three homo-dimer complexes and three hetero-dimer complexes, each with different photophysical properties.^260^ The proportion of each homo-dimer species can be interrogated by using absorbance and fluorescence at distinct wavelengths, offering a data-rich output from a single mixture. By monitoring these changes in response to analytes that interfere with the self-assembly process, and analysing the results using principal component analysis, individual serum albumin proteins could be discriminated. The authors demonstrated the real-life applicability of this approach by identifying species of fish from protein mixtures, outlining the use of such a technology in food security. Whilst this approach is yet to be commercialised to the best of our knowledge, the operational simplicity and information richness of this single dynamic mixture will likely see use across a range of applications.^260^

Detection of anions is of great importance for biomedical, environmental and industrial applications,^261^ and has thus been the subject of extensive investigation by the supramolecular community.^262^ The immobilisation of sensors into materials is often essential when considering the translation of these technologies into real-world devices; this can be achieved either through covalent attachment, risking decreased sensitivity and increasing synthetic complexity, or through non-covalent encapsulation which can result in the leaching of the sensor. To navigate these issues, Willcock and co-workers have developed a dual-encapsulation strategy, encapsulating a europium-based luminescent anion sensor within polymeric particles,^263^ which are in turn embedded within a 2-(hydroxyethyl)methacrylate hydrogel matrix.^264,265^ The polymer particles retain the same affinity for the bicarbonate anion as the molecular europium complex, indicating that the presence of the polymer does not inhibit the diffusion of analytes. When the particles were embedded into the hydrogel no leaching of the encapsulated sensor was observed over two months, with retention of reversible bicarbonate detection. This method presents a potential platform for the incorporation of a range of supramolecular sensors into materials to aid device design.

**Fig. 31:**
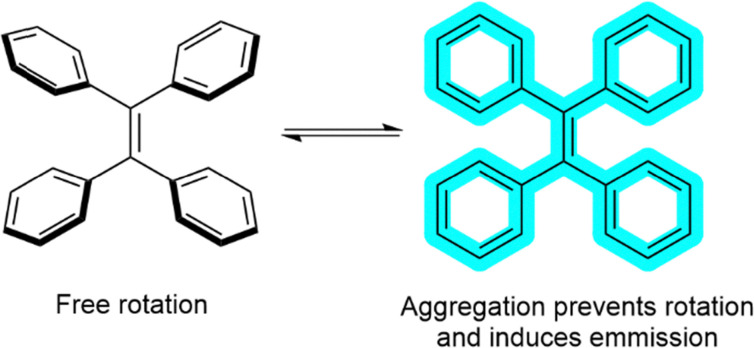
The aggregation induced emission phenomenon exemplified by tetraphenyletheylene.^266^

Aggregation induced emission (AIE) is a phenomenon observed in which the fluorescence of some molecules is weak in dilute solutions but increases upon aggregation due either to decreasing solubility or increasing concentration. This effect arises from the ability of these AIE luminogens (AIEgens) to relax from their excited state to their ground state *via* non-radiative processes in solution, a process which is prohibited upon aggregation.^266^ The mechanism for this process is generally associated with the restriction of molecular vibrations or rotations, for example in the rings of the classic AIEgen tetraphenylethene, Fig. 31. Their intermolecular interactions with other molecules and their environment offers this technology a variety of uses across a range of fields, such as biological imaging.

**Fig. 32:**
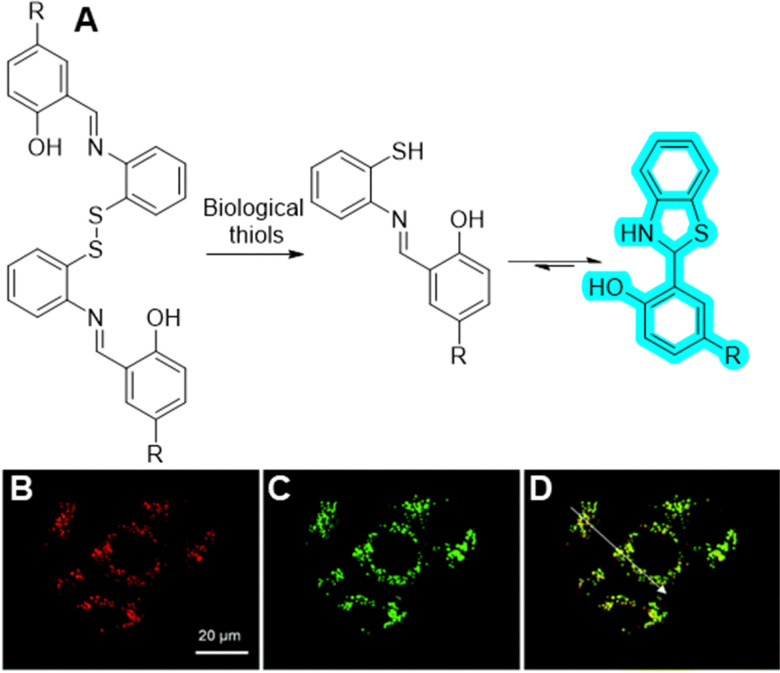
(A) The structure and activation of the bis(2-(2-hydroxybenzylidene)amino)aryl disulfides by biological thiols to form fluorescent AIEgens. Fluorescent microscopy images of HeLa cells treated with (B) photoactivated AIEgen (50 μM), (C) BODIPY493/503 and (D) a merged image of the two. Reproduced with ref. 267 and 268 under creative commons license CC BY 4.0.

An example for the use of AIEgens in bioimaging are the bis(2-(2-hydroxybenzylidene)amino)aryl disulfides.^267,268^ These probes feature targeting units, causing them to accumulate in their lipid droplets (R = OMe) or in acidic lysosomes (R = morpholine). Under biological conditions a S–S reduction occurs yielding the Schiff-base adduct which can then undergo a photooxidativedehydrogenation to form AIEgen 2-(2-hydroxyphenyl)-benzothiazoles. These probes were then shown to be capable of imaging individual organelles within live HeLa cells (Fig. 32), capable of both one- and two-photon excitation, demonstrating the power of AIEgens in bioimaging.

**Fig. 33:**
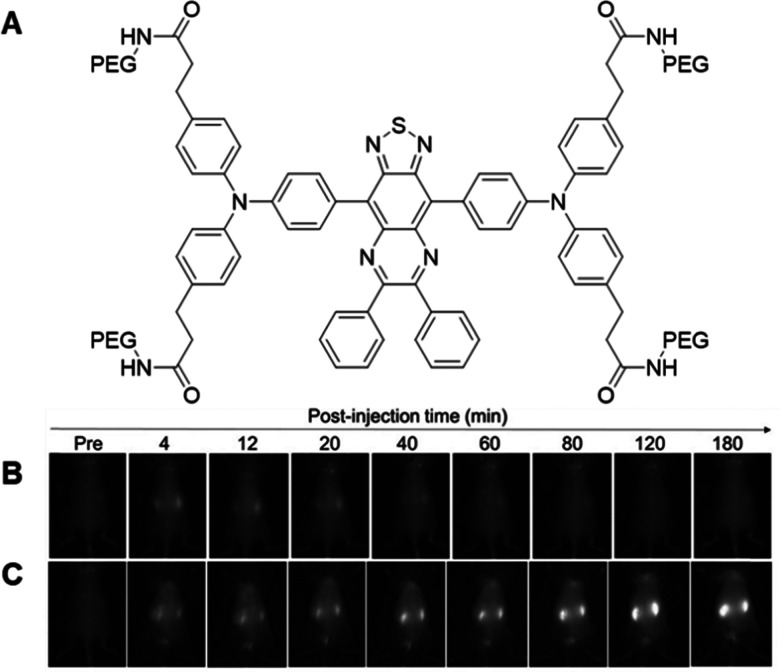
(A) The structure of the AIEgen developed by Yan and co-workers for imaging renal fibrosis. Live mouse fluorescence image of healthy mice (B) and those with induced renal fibrosis (C) after injection of AIEgen. Reproduced from ref. 271 and 272 with permission from Wiley, copyright 2022.

Beyond imaging of cells *in vitro*, AIE has also been applied to diagnostic tools. Left unchecked, chronic kidney disease has the potential to progress towards major kidney disfunction, however due to the latency of the disease it can be hard to diagnose and administer appropriate interventions in a timely manner.^269^ One of the pathophysiological presentations of chronic kidney disease is renal fibrosis, which can provide a good indicator of disease progression, however the current gold standard for estimating the progression of this fibrosis is histological examination of renal biopsies.^270^ This is an invasive and time-consuming procedure, bringing with it risks of bleeding and infection, and only examines a small section of the kidney which may fail to capture the bigger picture. To overcome these issues, Tang and co-workers developed a water soluble AIEgen capable of the real time differentiation of fibrotic and healthy kidney tissues *in vivo.*^271,272^ Their lead compound (Fig. 33A) featured an electron deficient core with highly twisted 3D character, flanked by two-electron rich triphenylamine groups to introduce charge transfer character.^271^ Appended to these terminal groups were short chain carboxylic acids to enable further functionalisation with PEG, endowing the system with water solubility. These compounds could be injected *in vivo* and showed rapid (4 minute) progression to the kidneys, after which they were excreted. However, in diseased fibrotic kidney tissue (folic acid-induced) the signal continued to grow for three hours due to slowed metabolism *via* urinary excretion, producing high-definition images of the affected areas in live mice (Fig. 33B and C).

#### Supramolecular applications of photoswitches

Molecular photoswitches are compounds which undergo reversible photoreactions, often an *E*–*Z* isomerisation around C=C (stilbenes,^273^ hemiindigo,^274^ and indigo),^275,276^ C=N (hydrazones,^277^ imines)^278^ or N=N (aryl/heteroaryl azo-compounds)^279^ bonds, or cyclisation reactions (donor acceptor Stenhouse adducts, diaryletheylenes). These reactions are often associated with a concomitant change in molecular shape, size and topological properties, which has thus led to their development in a number of fundamental supramolecular systems. Whilst most of these studies are in their infancy, there are a rising number of practical photoswitchable supramolecular systems.

#### Supramolecular sensing using photoswitches

##### Commercial case study: Optosense

**Fig. 34:**
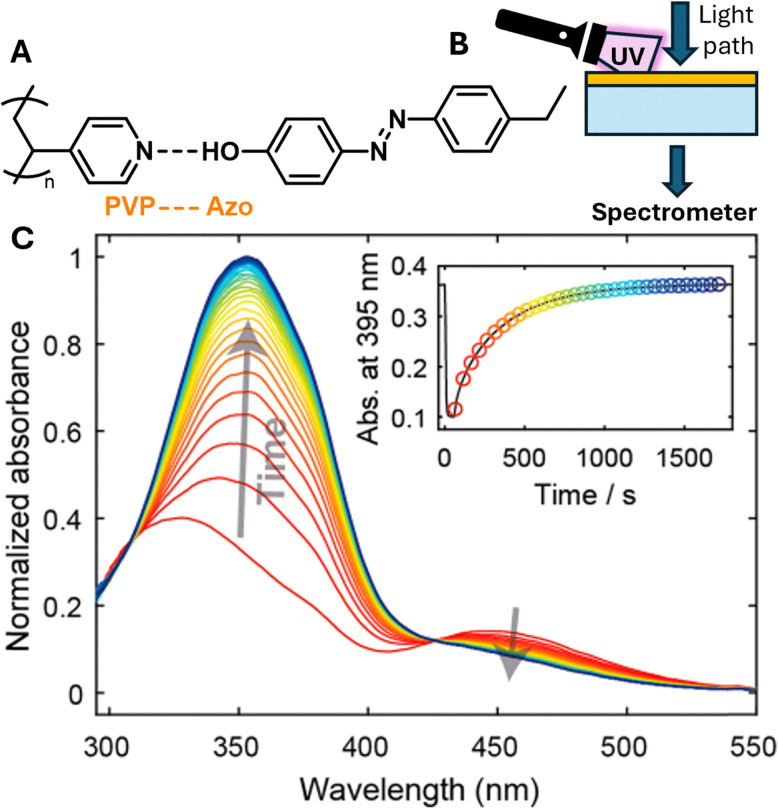
(A) Hydrogen bond mediated complexation between poly(4-vinylpyridine) and a hydroxy azobenzene. (B) A schematic representation of the measurement set up used to measure the changing rate of thermal reversion; the orange bar represents a thin film (10 μm) of PVP-azo which is placed on top of a transparent substrate. (C) A typical thermal reversion measurement of the *cis* to *trans* isomerisation of an azobenzene measured in the set up. Adapted from ref. 280 and 281 under creative commons license CC BY 4.0.

Many photoswitches display properties (*i.e.* onset wavelength, thermal half-life, quantum yield) that are dependent on their environment, commonly a solvent. 4-Hydroxyazobenezenes have a thermal half-life that can change up to five orders of magnitude in the presence of polar solvents due to tautomerization mediated by hydrogen boding. Making use of these effects, the Priimagi group developed a sensor capable of detecting vapours of polar solvents (*i.e.* humidity) using 4-(4-ethylphenylazo)phenol embedded in a solid poly(4-vinylpyridine) (P4VP) matrix.^280,281^ The pyridine within the matrix is able to act as a hydrogen bond acceptor, enabling efficient loading of the hydroxyazo compounds (Fig. 34). Current commercial relative humidity sensors function by measuring a secondary output triggered by adsorption of water molecules to a sensing material; this brings with it geometric considerations and the need for calibration. Conversely, this system measures the change in isomerisation kinetics directly circumventing these issues. The fabricated sensor showed comparable results to a commercial hygrometer and could detect a change in relative humidity of 20% to 70% in 11 seconds, and back from 70% to 20% in 15 seconds. The technology, termed Optosense, has a multitude of potential commercial uses such as packaging and construction, and is inviting collaboration with other industries.^282^ The authors have since investigated other hydroxyazobenes and polymer matrices to uncover methods to tune properties such as activation rate towards the specific applications.^283^

#### Vapour sensing and actuation

Sensing water vapours and volatile organic compounds (VOCs) plays a significant role in monitoring air quality, identifying hazardous leaks or emissions in industrial plants, or optimising the conditions for crops growth in agriculture. Also relying on sensing, actuation is the process of converting a stimulus or input (such as electrical, thermal, chemical, or environmental signals) into mechanical motion, and paves the way to applications in soft robotics, adaptive materials, and energy harvesting.^284^ These devices currently face challenges such as limited selectivity, size and cost constraints, and restricted environmental operational ranges and degradation, which hinder their robustness and widespread practical application. Employing host/guest supramolecular systems has the potential to overcome these limitations, since these systems can be tuned to ensure the reversibility and selectivity of the vapour absorption process, as well as high sensitivity.

**Fig. 35:**
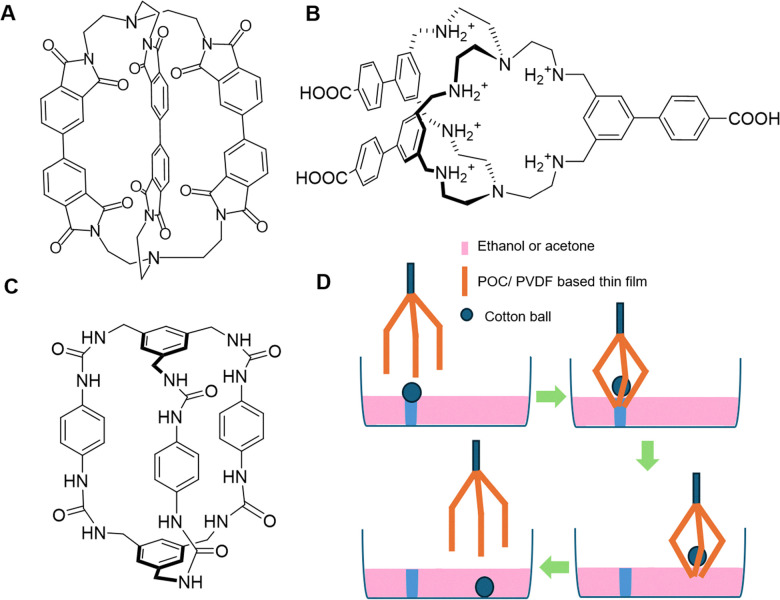
Chemical structures of the POCs used as sensors for humidity ((A) ref. 285 and (B) ref. 286) and organic vapours ((C) ref. 287). (D) A general schematic showing how a POC-based robot could grip and then release a cotton ball by having three thin films twisting around it when immersed in a chamber of either acetone or ethanol.^287^

For example, in 2023, Zhang and co-workers produced a humidity-responsive actuator, using a porous organic cage in a polymer matrix (Fig. 35A).^285^ This innovative device converts atmospheric moisture into mechanical movement, capable of powering grippers or robotic components. When integrated with a piezoelectric film, this innovation can also generate electricity. This marks the first instance of a porous molecular cage being utilized for such purposes, paving the way for advanced smart materials and novel energy harvesting techniques.

Similarly, in 2024, Khashab and co-workers developed a moisture sensor using a POC^286^ (Fig. 35B) that forms water channels upon water adsorption. The sensor's mechanism relies on structural changes in the material's pores. Molecular dynamics simulations further supported the formation of water channel structures under high moisture conditions. Here the innovators successfully applied this technology to a touchless screen operated by finger gestures, which paves the way to building new sensor-driven appliances. However, although these results open doors to new applications of similar supramolecular host–guest systems towards water sensing,^288^ challenges towards translation remain, such as the moisture level of fingers being different in different geographical regions and seasons, and the distance between the operating finger and the sensor was limited to 5 mm.

Later that year, the same group developed an innovative organic vapour-triggered actuator using urea-based POCs^287^ (Fig. 35C). This actuator combines a urea cage composite with a piezoelectric polyvinylidene fluoride (PVDF) matrix, enabling solvent-responsive mechanical motion and energy harvesting. Here the urea cage's reversible polymorphic transformations, driven by selective host–guest interactions with organic vapours, result in specific distinct mechanical deformations (*i.e.* bending and twisting), based on the solvent's molecular structure, while the PVDF matrix converts the motion-induced strain into electrical energy, allowing self-powered operation. The team demonstrated the actuator's practical application by constructing and testing a multi-tasking soft robot, which can autonomously navigate vapour gradients (Fig. 35D). This robot could grip and then release a cotton ball by having three thin films twisting around it when immersed in a chamber of either acetone or ethanol, caused by the different packing of the host/guest complex. When the soft robot is removed from the medium, the claws release the cotton the ball and straighten, as the vapours diffuse out of the POC cavities.

### Metallurgy

Supramolecular recognition has underpinned approaches to isolate precious and industrially useful metals for several decades.^289^ Since 2021 we have seen significant advances in both liquid-phase extraction methodologies, alongside processes which move away from traditional liquid phase binding, including selective precipitation and extractant immobilisation in resins and gels. It has been suggested by some authors that the latter approach can aid the re-isolation and re-use of the supramolecular extractants, potentially yielding more economical and thus industrially viable processes^290^ – in addition, the recyclability of the metals themselves has become an increasingly important consideration, given that many widely used metals are becoming more and more scarce and difficult to procure.^291^

#### Gold

Stoddart and colleagues have pioneered workflows centred on cyclodextrins for the recovery of gold from ores and electronic waste, in the form of alkali metal haloaurate salts. Their earlier work in this area, and the creation of Cycladex Ltd in 2014, discussed in our earlier review,^292^ established the use of α-CD for the spontaneous precipitation of an extended {[K(OH_2_)_6_][AuBr_4_]⊂(α-CD)_2_}_*n*_ chain superstructure from water followed by a workflow to isolate metallic gold and recover the α-CD.

**Fig. 36:**
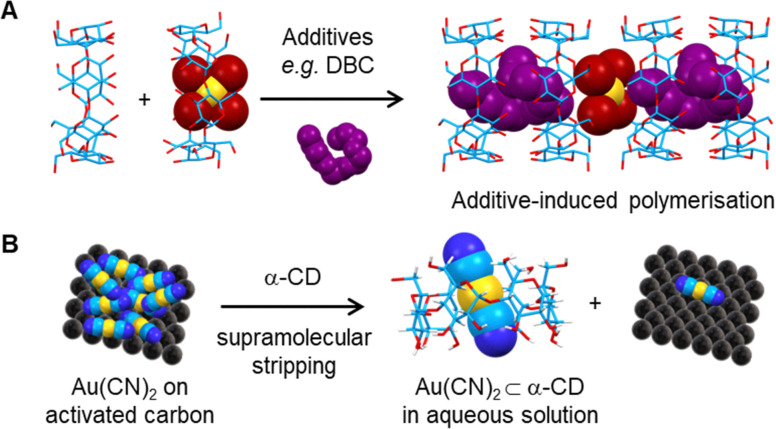
Gold recovery processes using cyclodextrins: (A) additive-induced supramolecular polymerisation, in which the addition of an additive causes the precipitation of gold-containing solids;^293,294^ (B) a schematic representation of the supramolecular stripping process to remove immobilised Au(CN)_2_^−^ from the surface of activated carbon.^295^

This group subsequently developed an additive-induced supramolecular polymerisation process using β-CD for gold recovery from gold-bearing scrap.^293,294^ They found that AuBr_4_^−^ can bind with β-CD, initially yielding a soluble host–guest complex with a binding constant of 4.5 × 10^4^ M^−1^ determined by NMR titration in D_2_O. Crucially, the authors discovered that using additives such as dibutyl carbitol (DBC) appears to drive a change in binding mode that leads to supramolecular polymerisation and subsequent precipitation – thus providing a means to isolate the host–guest complex from solution. Solid state analysis revealed that in the initial complex, the AuBr_4_^−^ is localised within the cavity of the β-CD, stabilised by multiple weak [C–H⋯Br–Au] hydrogen-bonding interactions and the hydrophobic effect. After the addition of a DBC, the resulting cocrystals include DBC coils, which are contained within and bridge two β-CD cavities (Fig. 36A). This pushes the AuBr_4_^−^ out of the inner cavity, causing it to localise between the primary faces of the β-CDs, thus creating supramolecular cross-links which facilitate the formation of extended, one-dimensional nanostructures, that trigger precipitation and enable recovery. The authors reported a protocol to recover gold from gold-bearing scrap with gold recovery efficiencies up to 99% and high selectivity.

In addition to the work on recovering haloaurate salts, Stoddart and Liu have also focussed their attention on the linear Au(CN)_2_^−^ anion. Commercial gold mining processes use activated carbon to separate dissolved Au(CN)_2_^−^ from leached pulps;^296^ following this, harsh conditions are typically used to strip the Au(CN)_2_^−^ from the activated carbon for further processing.^297–299^ As such, Au(CN)_2_^−^ is arguably the most relevant anion in the gold-mining industry and more economical methods to extract it from activated carbon surfaces are sought after.

In 2020 Liu, Stoddart and co-workers first published their finding that α-CD can function as a molecular receptor for the linear Au(CN)_2_^−^ anion which is capable of stripping it from the surface of activated carbon,^295^ and this innovation has led to the publication of a new patent in 2024.^300^ They found that α-CD forms a 1 : 1 complex with Au(CN)_2_^−^ in D_2_O, and determined a *K*_a_*via* NMR titration of 8.1 × 10^4^ M^−1^. The single crystal X-ray structure of [Au(CN)_2_^−^⊂α-CD], (shown in Fig. 36B), demonstrates that the linear Au(CN)_2_^−^ anion can be threaded through the cavity of the α-CD, with the structure stabilised by multiple [C–H⋯π] and [C–H⋯anion] interactions. The authors exploited the strong binding to develop a “supramolecular stripping” protocol to remove K[Au(CN)_2_] from the surface of activated carbon. KAu(CN)_2_-loaded carbon was treated with an aqueous solution of α-CD at room temperature, resulting in the formation of [K^+^][Au(CN)_2_^−^⊂α-CD] in solution that could be isolated from the activated carbon by filtration. Inductively coupled plasma mass spectrometry revealed that the recovery of Au increases as the concentration of α-CD increases. The 2024 patent covers both the supramolecular stripping methods and subsequent processing to enable gold recovery using methods such as electrolysis. The authors propose that these protocols can significantly reduce the costs, energy consumption and environmental impact of commercial gold mining.

Love, Morrison, and co-workers have found great success in the use of simple amide ligands for the recovery of gold from electronic waste. Following on from their previous studies which focussed on liquid–liquid extraction approaches, in 2021 these authors reported a simple, tertiary diamide ligand able to selectively co-precipitate with AuCl_4_^−^ from aqua regia solutions of electronic waste.^301^

**Fig. 37:**
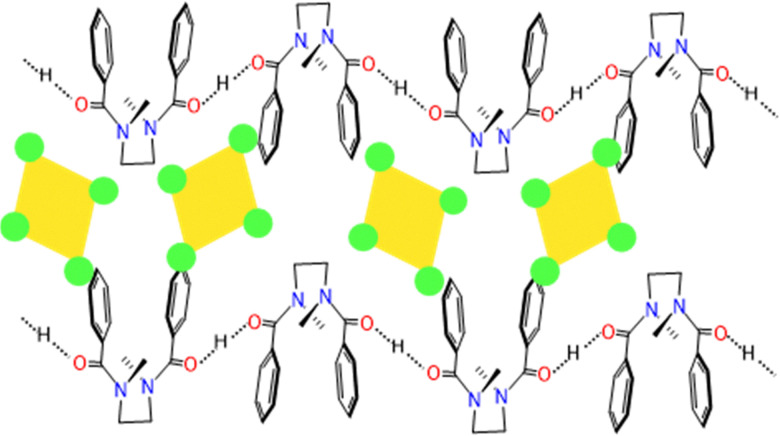
A schematic depiction of the solid-state structure of [HPAL][AuCl_4_], a gold containing precipitate enabling the isolation of gold from electronic waste.^301^

Initial experiments demonstrated that the polyamide ligand (PAL) shown in Fig. 37 could facilitate the liquid–liquid extraction of gold from 2 M and 6 M HCl into chloroform, but the authors noted the formation of gold-containing precipitates. They therefore investigated the use of PAL as a precipitant for gold salts. The structure of [HPAL][AuCl_4_] was determined by single crystal X-ray analysis (Fig. 37), which showed that ligand PAL associates into chains bridged by an intermolecular hydrogen “chelate” between amide units, while the electron rich aromatic rings enable face-to-face π-bonding with the AuCl_4_^−^ anion. Adding a 10-fold excess of PAL to solutions of HAuCl_4_ in 2 M or 6 M HCl in the absence of an organic solvent led to the formation of yellow precipitates which contained >99% of the gold in the system depending on the initial gold concentration. Gold precipitation could also be achieved starting from solutions in aqua regia or 2 M H_2_SO_4_, making the process viable under testing conditions. Simply washing the precipitate with water led to the release of HAuCl_4_ into aqueous solution, allowing the gold to be isolated and the ligand to be regenerated for further use. The authors found that the uptake of gold was highly selective using a stochiometric quantity of PAL, with minimal (<5%) co-precipitation of other metals from gold-containing, mixed-metal solutions in 2 M HCl. Using excess PAL in 6 M HCl solution could further enable the uptake of Fe, Sn and Pt, likely due to the enhanced concentration of the chloridometallates under these conditions, which shows that PAL could potentially be used to sequentially remove gold and then other metals from feed streams.

Following this initial report, the authors published a patent on the selective precipitation of metals using amide compounds in 2023, covering the use of a range of similar diamide ligands for this purpose,^302^ and in 2024 published an additional paper reporting insight into the unexpectedly favourable and selective precipitation process,^303^ which suggests that the precipitation is thermodynamically driven and that the observed selectivity is due to the gold precipitate being amongst the most thermodynamically stable structures at room temperature.

#### Rhodium

Rhodium is a high value platinum group metal (PGM) with notable uses in chemical catalysis, electronics,^304^ and jewellery. Its extraction from both ores and secondary sources (such as recycled catalytic converters) is complicated by the presence of iridium, as there are few effective processes for selectively separating these species in acidic solution, given that both metals are in the same group in the periodic system and show similar structural properties. An additional complication is the presence of numerous rhodium metallate species [RhCl_*n*_(H_2_O)^6−^_*n*_]^(*n*−3)^ (*n* = 0–6) in solution, which makes it challenging to design a single reagent that can selectively extract all the rhodium from a solution.

**Fig. 38:**
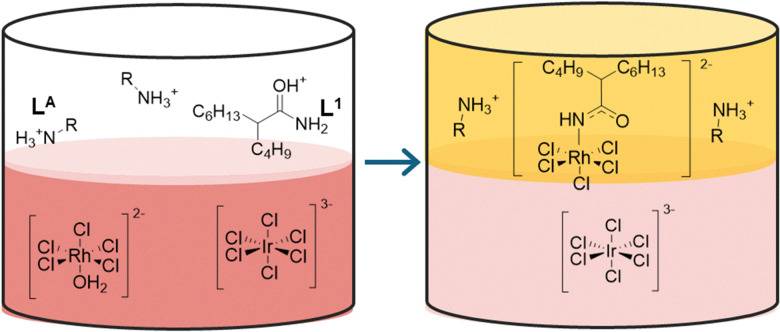
A schematic depiction of the synergistic liquid–liquid extraction of rhodium ions from iridium-containing mixtures. L^A^ = Primene^TM^ 81R, a mixture of isomers with R = C_12–14_H_26–30_.^305,306^

Following their success in using lipophilic amides in gold extraction processes, Morrison and Love have published and patented methods for the liquid–liquid extraction of rhodium from iridium.^305,306^ In earlier work,^307^ these researchers showed that a combination of a primary amide (L^1^, Fig. 38) and a branched primary amine (L^A^, Fig. 38) can synergistically function to extract rhodium chlorometallate complexes from HCl solution into organic solvents such as toluene. This combination of ligands was found to extract a mixture of both [RhCl_6_]^3−^ and [RhCl_5_(L^1^)]^2−^ metallates into the organic phase, evidenced by UV-vis spectroscopy and electrospray ionisation mass spectrometry measurements. In contrast, extraction using L^A^ alone only resulted in the extraction of [RhCl_6_]^3−^, thus demonstrating that the synergistic action of both ligands is required for the extraction of [RhCl_5_]^2−^ anions. The authors proposed that [RhCl_6_]^3−^ was rapidly extracted by an outer-sphere ion pair mechanism involving L^A^ only, whereas [RhCl_5_]^2−^ (which was extracted more slowly) required an inner sphere mechanism, with L^1^ displacing a water or chloride ligand to form a [RhCl_5_(L^1^)]^2−^ complex. Based on the NMR analysis, the amide ligand L^1^ was suggested to tautomerise to the enol form and coordinate to rhodium through the nitrogen atom as shown in Fig. 38. The initially reported synergistic extraction system could extract >85% of rhodium from 4 M HCl.^307^ Following this, recent advances have involved the optimisation of L^A^ to improve its recovery, improve the selectivity over iridium, and develop a protocol to strip the extracted rhodium from the mixed ligand system.^306^ Although this new protocol does not enable the co-extraction of [RhCl_6_]^3−^, and hence the recovery of Rh is reduced, the process overall is more selective and industrially relevant, considering that the extractant can be re-used in multiple cycles.

#### Platinum

Stoddart and co-workers have also turned their attention to the selective isolation of platinum. They have established a selective separation of hexachloroplatinate anions, with advances published in 2021^308^ and a subsequent patent in 2024.^309^ Their processes are underpinned by their discovery that CB[6] macrocycles can bind to [PtCl_6_]^2−^ anions in acidic solution, triggering the rapid and spontaneous formation of a supramolecular crystalline material identified as CB[6]·H_2_PtCl_6_. Single crystal X-ray analysis revealed that there is a 1 : 1 stoichiometry between the CB[6] and the [PtCl_6_]^2−^ anions, and revealed that the anion does not reside in the macrocycle cavity but instead interacts with the outer surface *via* [Pt—Cl⋯H—C] hydrogen bonds and [Pt—Cl⋯C=O] ion-dipole interactions. Each [PtCl_6_]^2−^ anion has close contacts with six surrounding CB[6] molecules. This process was found to be selective for [PtCl_6_]^2−^ over the similar anions [PdCl_4_]^2−^, [PdCl_6_]^2−^ and [RhCl_6_]^3−^ as none of these anions triggered a spontaneous crystallisation or precipitation process. The authors developed a workflow by which platinum metal could be isolated from mixtures of platinum, palladium and rhodium (representative of the mixed metal waste found in spent catalytic converters^310–312^). The addition of CB[6] triggered the precipitation of CB[6]·H_2_PtCl_6_ microcrystals which could be isolated and dispersed in an aqueous solution. The addition of hydrazine reduced the [PtCl_6_]^2−^ to black metallic platinum and regenerated the CB[6] for recycling.

#### Lithium

Lithium has seen a growing demand in the previous decade mostly due to its usage in batteries for electronic devices and vehicles.^313^ Since 2021, Sessler and colleagues have led several key advances in using supramolecular approaches to isolate lithium salts from mixed ion brines. Following earlier work which demonstrated the use of strapped calix[4]pyrrole derivatives for ion pair recognition and extraction,^314^ a 2021 paper^290^ and 2023 patent^315^ have reported the immobilisation of a strapped calix[4]pyrrole within a polymer matrix for LiCl separation. Their aim in developing polymer-supported hosts was to create a straightforward process by which to isolate their intended target and recycle their extractant.

**Fig. 39:**
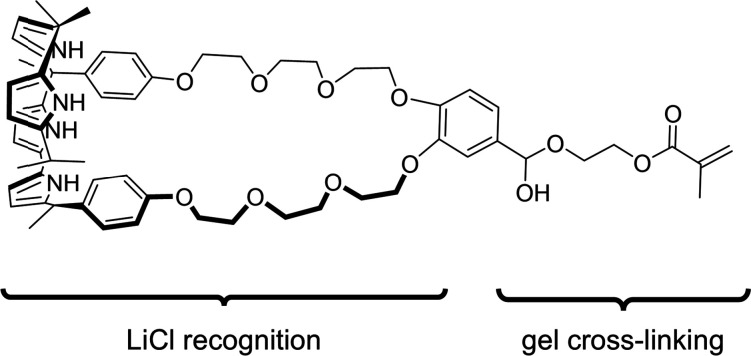
The structure of the strapped calix[4]pyrrole reported by Sessler,^290^ which contains a methacrylate unit for cross-linking into a polymeric gel.

They produced two strapped calix[4]pyrroles, including the example shown in Fig. 39, equipped with methacrylate handles to enable later copolymerisation with acrylate monomers and cross-linkers. Initial solid–liquid extraction (SLE) experiments in nitrobenzene-*d*_3_ and CD_3_CN found that significant changes to the NMR spectra of the monomers were observed upon exposure to solid LiCl that were consistent with binding a Li^+^ cation in the crown ether “strap” along with a Cl^−^ anion within the calix[4]pyrrole macrocycle. This binding mode was supported by single crystal X-ray crystallography. Competitive SLE experiments with mixtures of LiCl with NaCl, KCl, MgCl_2_ and CaCl_2_ indicated that both hosts displayed a preference for LiCl in CD_3_CN. However, no evidence of LiCl binding was observed in methanol-*d*_4_, which suggested that methanol could be a suitable solvent to remove bound LiCl from these hosts. The authors then explored the co-polymerisation of the strapped calixpyrrole with methyl acrylate (94.7 mol%) and 1,6-hexanediol diacrylate as a cross-linker (0.3 mol%) to produce an organogel. The internal solvent within the gels could be removed to yield swellable, solid macroscopic frameworks with solvent exchange capacity. Adding the solid polymer frameworks to saturated solutions of LiCl in acetonitrile led to the solid polymer swelling into a gel, and the uptake of dissolved salts evidenced by conductivity and inductively coupled plasma mass spectrometry analysis. The gels could then be physically picked up and removed, washed with acetonitrile and then placed in methanol which triggered the release of >96% of the LiCl into solution, enabling the LiCl to be isolated and the solid polymer to be regenerated by drying under vacuum for further use.

Building on this solvent-mediated “catch and release” system Sessler, Page, and co-workers explored the immobilisation of a neutral receptor on a polystyrene support for the capture of LiPF_6_ (currently the predominant lithium-based electrolyte in commercial lithium-ion batteries^316^) from a simulated electrolyte mixture.^317,318^ In this instance, an acyclic bis-dicyclohexylacetamide (BDCA) host was used for the capture of Li^+^ cations, reasoning that the concurrent uptake of PF_6_^−^ anions would maintain charge neutrality. The authors predicted that competitive anions such as fluoride and carboxylates would be less likely to be extracted due to the lower ion-pair energy expected for LiPF_6_ thus yielding anion selectivity without specific incorporation of an anion recognition site.

NMR spectroscopy titration experiments indicated that solution-phase BDCA bound Li^+^ strongly in CD_3_CN (*K*_a_ = 2.6 × 105 M^−1^) but that the affinity in methanol-*d*_4_ was significantly reduced. The BDCA was immobilised on a polystyrene resin with surface-bound NH_2_ groups *via* a flexible, *N*-hydroxysuccinimide linker. Conductivity measurements indicated that the BDCA-loaded resin could take up LiPF_6_ from MeCN solution with >90% of this ion pair being taken up from a 1.55 mM solution within 4 hours. Solid state NMR analysis confirmed the appearance of PF_6_^−^ anions bound to the resin. Transferring the loaded resin to anhydrous methanol led to the release of LiPF_6_ into solution, allowing isolation of the salt and recycling of the resin. They found that the resin could be re-used for at least five cycles with no significant decrease in the catch and release efficiency.

Combining insight from the previous studies, Page and Sessler have recently been expanding the scope of metal-recovery using immobilised receptors, reporting that BDCA ligands can be 3D-printed into supramolecular polymer sorbents for cobalt recycling.^319^ A key challenge they aimed to overcome was the capacity-flux paradigm, namely that supported host extractants typically suffer from low capacity (*e.g.* gels and polysterene beads) or require high pressures to achieve sufficient flux (*e.g.* nanoporous inclusions in granular separation systems). Here the authors developed a 3D printing approach to manufacturing microstructured sorbent materials with multi-scale geometric control, which has been reported to yield high performance materials for a range of separations.^320^

To achieve this goal, the authors designed BDCA hosts with a methacrylate functional group to facilitate co-polymerisation. They then adapted polymerisation-induced phase separation (PIPS) and digital light processing approaches, using tripropylene glycoldiacrylate (TPGDA) as the support material, 1-decanol and cyclohexanol as porogens, and (2,4,6-trimethylbenzoyl)phosphine oxide as a photoinitiator. These components could be 3D-printed using a layer-by-layer stereolithography technique and irradiated *via* LED exposure to produce printed objects with defined microstructures, which could be soaked in ethanol to remove unreacted monomers and porogens. They produced a range of different microstructures with different lattice geometries and pore sizes.

Having previously used BDCA-loaded materials for Li^+^ capture, here the authors exploited the similarities between the binding preferences of Li^+^ and Co^2+^ and used their BDCA-loaded polymers to extract Co^2+^. The Co^2+^ uptake properties of the 3D printed objects were evaluated by placing them in containers with solutions of CoCl_2_ in a range of “green” solvents for 12 hours. Strong CoCl_2_ binding and uptake was observed in ethanol and isopropyl alcohol, with weaker binding in H_2_O, facilitating a catch and release extraction that could withstand five cycles. They also found that Kelvin lattices with larger pore size yielded a faster rate of uptake, and geometries with larger surface area-to-volume ratios had both higher capacity and faster uptake rates. This therefore demonstrates that the fine control of microstructure that can be achieved *via* 3D printing can have a significant impact on the overall performance of the materials.

### Extraction of industrially useful compounds

The extraction and purification of *para* and *ortho*-xylene is very important in the petrochemical industry; the former is the precursor of phthalic anhydride, which is an important precursor in synthesis, while the latter is used in the large-scale production of phthalic acid, widely used in the synthesis of plastics.^321,322^ The meta isomer is also an important precursor in organic synthesis. Traditionally, they are obtained *via* the methylation of toluene, which is a not a regioselective process, and then separated through a series of fractional distillations, which overall represents a costly and unsustainable method. By exploiting specific non-covalent, reversible interactions, supramolecular systems enable highly selective-liquid–liquid extractions of target analytes between immiscible liquid phases, offering greater efficiency and tunability than traditional methods for challenging separations in analytical and environmental chemistry.^321–324^

Towards this application, for a number of years, Khashab and co-workers have used macrocycle and cage architectures as building blocks to construct intrinsically porous materials, and many have found preliminary application in the separation of natural gas and benzene derivatives.^323^ A 2020 publication,^324^ followed by a 2021 patent,^325^ describes an innovative liquid–liquid extraction method using CB[7] to selectively separate *ortho*-substituted benzene compounds, with a focus on xylenes, for its isomers, which are notoriously difficult to purify by traditional distillation or crystallization approaches. Unlike solid–vapor adsorption techniques that require high temperatures and pressures, CB[7] enables separation under mild, ambient conditions.^326^

It was found that CB[7] binds *ortho*-xylene (OX) strongly, forming a 1 : 1 complex. NMR spectroscopy and isothermal colorimetry (ITC) confirm this selective binding, with a binding constant (*K*_a_) of 8.9 × 10^5^ M^−1^ for OX@CB[7]^−^ over 20 times greater than for *meta*-xylene (MX, 4.2 × 10^4^ M^−1^) and *para*-xylene (PX, 3.3 × 10^4^ M^−1^). This high affinity allows CB[7] dissolved in water at 4 g L^−1^ to selectively capture OX from a 1 : 1 : 1 mixture of xylene isomers. The complexed OX can then be efficiently extracted into an organic solvent, achieving purities exceeding 92% after a single extraction cycle. The authors found that the complexation process is enthalpy-driven, largely due to the release of water molecules from CB[7]'s cavity, which compensates for the entropic penalty. Kinetic studies reveal that OX@CB[7] complexes dissociate much more slowly than MX or PX complexes. DFT calculations further show that in aqueous solution, OX prefers a parallel orientation inside CB[7], maximizing favourable interactions and minimizing binding energy, a binding mode inaccessible to the other isomers due to their elongated shapes.

Extending beyond model mixtures, CB[7] was tested on industrial samples such as the C8 aromatic fraction from Pygas obtained from light Arabian crude oil, which contains 60–70% xylenes, ethylbenzene, and 30–40% styrene (ST). The C8 fraction is usually used for the gas phase isomerisation of xylenes to PX, but the process is slow and requires an advanced industrial setup.^327^ OX and ST show a small difference in boiling point of only 0.8 °C, which makes them hard to separate using traditional methods. Despite this, CB[7] achieved over 83% separation efficiency for OX, with the presence of ST having no adverse effect. Additional tests on crude industrial samples containing high levels of aliphatic hydrocarbons (*e.g.* cyclohexane and methylcyclohexane) showed that CB[7] could selectively extract these highly symmetrical spherical molecules due to their shape complementarity. However, such aliphatic content may reduce CB[7]'s selectivity for aromatic isomers, indicating that CB[7] is most effective for processed streams like the C8 aromatic fraction rather than unrefined crude oil. Therefore this technology offers several advantages over those that are currently used: it operates under ambient temperature and pressure, reducing energy consumption compared to traditional distillation; CB[7] is commercially available, chemically and thermally stable, and recyclable for at least five cycles without loss of selectivity. The approach provides exceptional specificity for the separation of OX – over 92% purity after one extraction from mixtures of isomers – and over 83% extraction efficiency from complex industrial mixtures. Overall, this research demonstrates the potential of stable supramolecular hosts like CB[7] to revolutionise energy-intensive industrial separations by combining high selectivity, efficiency, and sustainability.

### Remediation of environmentally harmful substances

#### Polyfluoroalkyl substances

As outlined in an earlier section, PFAS, or per- and polyfluoroalkyl substances, are synthetic compounds known as ‘forever chemicals’ due to their strong C–F bonds which make them resistant to degradation.^328^ Their persistence in the environment and potential health risks have raised significant concerns and led to increased regulation.^329^ In general, there are limited advances in technologies able to remove PFAS from the atmosphere, water and soil. Activated carbon adsorption, electrochemical oxidation or plasma-based destruction^330^ are examples of techniques used so far on large scale to break down PFAS, however they are all very energy-intensive and often lack adaptability to the diverse PFAS mixtures found in the environment. Structurally, PFAS are usually generated as a family of fluorinated carbon chains which include many different, volatile, small molecules, and are found in small concentrations in water and air, hence high selectivity and activity are required in order to successfully isolate and break them down.

**Fig. 40:**
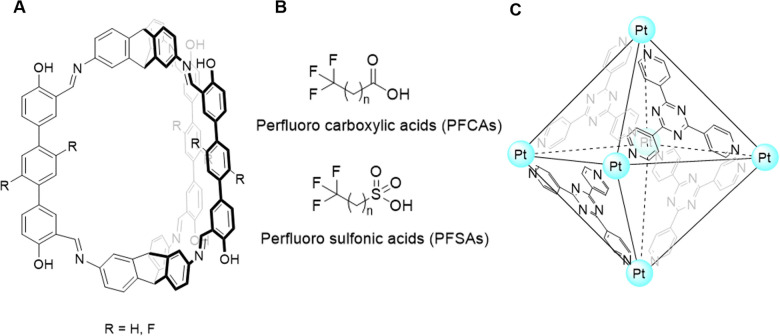
(A) Chemical structure of the POC for selective encapsulation of hydrocarbons (R = H, H-cage), or fluorinated compounds (R = F, F-cage);^331^ (B) general chemical structures of PFCAs and PFSAs, PFOS and PFOA are the structures corresponding to *n* = 7; (C) chemical structure of the MOC used to trap PFOS selectively.^332^

Finding a type of material that can be adapted for more chain lengths is very challenging, and this has been a significant focus in the supramolecular field in recent years. Mastalerz and co-workers developed a range of three isostructural imine-based POCs with various degree of fluorinated side-chains.^331^ They studied the gas adsorption of fluorinated alkanes in comparison to nonfluorinated gases, and found that the POCs with a higher content of fluorine showed increased preference for fluorinated gases (F-cage, Fig. 40), whilst those with no fluorine showed a higher preference over non-fluorinated gases (H-cage). Furthermore, the F-cage show excellent selectivity for PFASs *versus* other nonfluorinated gases. The cage was also found to be stable under a range of conditions, including in HCl_aq_ (0.1 × 10^−3^ M) or NaOH_aq_ (1 × 10^−3^ M) solutions for 24 hours, and recyclable for seven consecutive adsorption/desorption cycles, with the cage being stored for 204 days under ambient temperature between two gas adsorption cycles.

As some of the most produced and studied PFAS for decades, PFOS and PFOA (Fig. 40B) are long-chained, polar surfactants, capable of bioaccumulation and sorption, and can be transported through various modes of action in the environment, thereby posing toxicity to organism.^332^ In 2024, Elgrishi^333^ discovered the usage of previously well-known Pd-based metal–organic cage (MOC) pioneered by Fujita^334^ (Fig. 40C) that can selectively trap PFOS over other anions commonly found in drinking water, such as nitrate, fluoride, chloride, phosphate, or sulfate. The MOC's large hydrophobic cavity can accommodate two PFOS molecules, as indicated by NMR data, and up to 12 PFOS molecules can precipitate as an aggregate with the cage at higher concentrations. Despite its strong affinity for PFOS, the MOC can be recycled and reused, enabling a protocol to concentrate PFOS in organic solvents for further processing or degradation. In the same year, Chi and Sessler reported the usage of a novel non-porous adaptable crystal (NAC) based macrocyclic organic capsule^335^ capable of intaking PFOA through five reuse cycles, that could be recovered by heating at 80 °C under vacuum. Both papers mention that however, further research is needed to develop effective PFOS and PFAS degradation methods.

Similarly to PFAS, SF_6_ is notorious for its chemical and thermal stability. Studies have estimated that the total greenhouse impact of SF_6_ on the environment is around 24 000 times higher than that of CO_2_.^336^ In contrast to PFAS, SF_6_ recycling is preferred to degradation, as it can then be reused as a cooling agent.^337^ For this purpose, supramolecular cages, such as a porous organic cage based on CC3^338^ (Fig. 26A) have been studied briefly towards this scope, and could potentially find usage for purifying water from industrial waste.

#### Carbon dioxide (CO_2_)

**Fig. 41:**
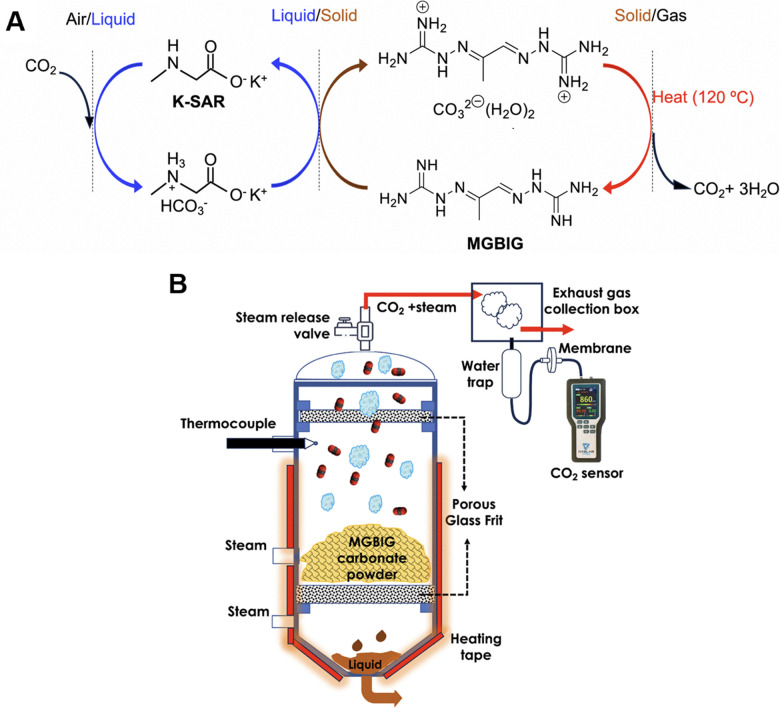
(A) A 3-step CO_2_ capture and release cycle based reported by Custelcean and co-workers based on the crystallisation of MBIG carbonate; (B) a schematic illustration of a continuous-flow direct-steam sorbent regeneration (DSR) protocol, reported to increase the efficiency of step iii) in part (A). Reproduced from ref. 345 in line with Elsevier's STM Permission Guidelines (2024).

In recent years, Custelcean and co-workers have led research into the direct air capture of CO_2_, aiming to develop chemical processes to remove CO_2_ from the atmosphere in line with global decarbonisation efforts. Significant challenges to overcome in this quest have been the low atmospheric concentration of CO_2_ (and hence the need for strong and selective CO_2_ binding), and the high energy input required to regenerate sorbent materials. In their earlier work, Custelcean's team developed stepwise processes by which CO_2_ can be directly sequestered from air, involving: (i) the conversion of CO_2_ into soluble carbonate or bicarbonate salts assisted by interaction with amino acids or short peptides; (ii) co-precipitation with bis-guanidine sorbents through selective anion recognition, effectively sequestering a CO_2_-containing material which can be removed from solution by filtration; (iii) regeneration of the sorbent by heating and releasing the CO_2_.^339–344^ A representative process is illustrated in Fig. 41A, in which an amino acid-derived salt K-SAR is used to initially stabilise the sequestered CO_2_ as HCO_3_^−^ in aqueous solution, which is then bound and precipitated as the CO_3_^2−^ salt by MGBIG, an iminioguanidinium anion sorbent.^345^

The authors’ most recent advances in this field have focussed on step (iii): a 2024 paper^345^ and 2025 patent^346^ report an energy-efficient, steam-stripping method to regenerate bis(guianidinium) sorbents such as MGBIG from their carbonate salts and re-isolate the sequestered CO_2_ (Fig. 41B). In this process, the solid MBIG carbonate is positioned between two porous frits at the top and bottom of a reactor to enable the release and collection of gases and liquids respectively. Steam is fed into the reactor to heat the loaded powder and trigger CO_2_ release. Molecular dynamics simulations indicate that the rate of heat transfer between steam and the solid sorbent is greater than between the solid and air, which means the steam-stripping process is more efficient than heating through conventional means. The authors found that this type of protocol reduces the overall CO_2_ capture cost by 50% compared to traditional conductive heating methods and consequently strengthens the industrial credentials of this carbon capture and release chemistry.

#### Anions

**Fig. 42:**
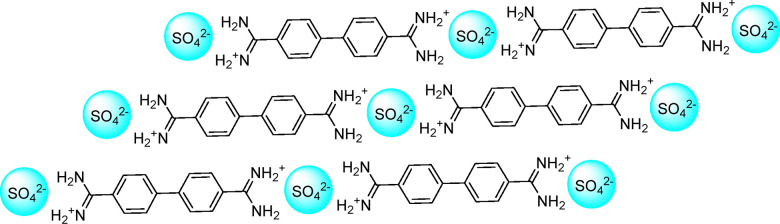
Chemical representation of the sheet-like structure of the bis-amidinium sulfate precipitate reported by White and co-workers.^349^

Sulfate (SO_4_^2−^) extraction remains a hot topic in the community and has been extensively studied^347^ and referenced before.^292^ Sulfate removal is critical in nuclear waste disposal primarily due to its detrimental effects on vitrification processes and long-term waste stability.^348^ Given their hydrophilic nature, sulfate ions are hard to remove from water with high selectivity towards other similar oxoanions. There have been recent advances, such as the gram synthesis of a bis-amidinium compound by White and co-workers in 2023,^349^ which can undergo anion exchange with chloride ions, precipitating 2D coordination sheets (Fig. 42). As shown by single crystal X-ray diffraction, in the crystal structure, each sulfate anion receives eight charge-assisted hydrogen bonds from the amidinium N. This crystalline framework can be achieved using sulfate concentrations as low as 1 mM, demonstrating complete specificity against monovalent anions while maintaining high selectivity even toward carbonate (CO_3_^2−^) and phosphate (HPO_4_^2−^) ions. This system achieves over 90% sulfate removal in both seawater and highly acidic conditions typical of mining wastewater streams.

Similar tetrahedral oxoanions can be challenging to remove from wastewater, as an excess can degrade water quality by disrupting ecosystems, endangering human health, and damaging the irrigation infrastructure. Natarajan and collaborators have designed a cleft receptor based on a foldameric *N*,*N*′-dimethyl-*N*,*N*′-diphenylurea scaffold,^350^ which forms a 3 : 1 host–guest complex with sulfate, phosphate and arsenate ions in solid state by hydrogen bonding. When using a competitive medium of DMSO/H_2_O 9 : 1, the receptor showed a strong and selective affinity for arsenate. When the counter cation used was tetrabutylammonium, the receptor was able to extract all three target anions into organic chlorinated solvents (CDCl_3_ and CD_2_Cl_2_ were both used for this purpose).

In 2025, Flood and co-workers have designed a light-responsive cyanostar macrocycle that was able to selectively extract PF_6_^−^ over Cl^−^, NO_3_^−^, and SO_4_^2−^, followed by photo-driven release (*via* stilbene isomerization) for quantitative recovery.^351^ As mentioned before when discussing PFAS, F-containing species can be challenging to isolate and purify especially from mixtures of other species. The method also successfully captured and released ReO_4_^−^ and radioactive ^99^TcO_4_^−^ at 90% efficiency, even at ultralow concentrations of four parts-per-billion. While reversibility remains limited, this proof-of-concept demonstrates light-controlled affinity switching for precise anion extraction and release between liquid phases. This is a particularly important innovation, as pertechnetate is highly radioactive with a long half-life, highly soluble, and mobile in water, leading to widespread environmental contamination and bioaccumulation risks. Its removal from nuclear waste and environmental mixtures is essential to prevent long-term ecological harm and ensure safe waste management.^352^

## Conclusions

Although our previous literature reviews didn’t use our patent searching methodology, over the past five years there has been a step-change in the drive towards, and the successful commercialisation of supramolecular innovations, as summarised in Table 1. It is also clear from the number and breadth of those commercialisation case studies communicated herein, that the successful translation of supramolecular innovations is not just located within a specific industrial sector, but across a highly diverse selection of sectors, which are of global financial, health and environmental importance *i.e.* pharmaceutical, medical device, cosmetics, batteries and solar energy. In addition, we also identify a plethora of supramolecular innovations where the inventors have successfully protected intellectual property claims through patenting their technology, demonstrating a clear intent to commercialize these inventions. This provides a substantial body of evidence towards the observation that there is a healthy innovation pipeline pump-primed for future translation within the international supramolecular chemistry community.

**Table 1:** Summary of key supramolecular translational innovations

Innovation	Function	Medium	Ref.	Patent(s)
CycloPure – DEXSORB®	PFAS removal	Solid	14, 15, 18, 353 and 354	US11155646B2
				US2024238761A
AgroFresh	Delay produce ripening	Liquid/gas	19 and 21	US5518988A
				EP1408752A2
Aqdot® – AqFresh™	Odour control	Liquid	22	EP3416693A1
Sparxell	Pigments	Solid	28 and 29	GB2610186A
Anthro Energy	Batteries	Solid	39, 41 and 42	US2022115692A
MOST	Energy storage	Solid/liquid	60–65	N/A
Mc-CDBA and Ca-CDBA	Sensor	Liquid	84 and 93	WO2025027606A1
Carbometrics and Zylo	Sensor	Liquid	95–100	US20150147275A1
				US10800747B2
Soluplus® drug solubilisation	Solubilisation agent	Liquid	108–114	US2018016144A1
				US20230037486A1
				US8999953B2
Innovotex	Drug carrier	Liquid	150–153 and 355	US10406167B2
Porous liquids	CO_2_ capture	Liquid	233–236, 240 and 241	US11717803B2
SmartWound®	Sensor	Solid/liquid	249, 251, 252 and 255	WO2025068709A1
DymerDyes	Sensor	Liquid	257–260	US11629128B2
AIE imaging	Diagnostics	Liquid	267, 268, 271 and 272	US12152010B2
				CN115490846B
Optosense	Sensor	Solid	280–282	US11391675B2
a-CD	Gold extraction	Liquid	295 and 300	US20240375084A1
PAL	Gold extraction	Liquid	301–303	WO2023007127A1
L^A^ and L^1^	Rhodium extraction	Liquid	305–307	GB2621920A
CB[6]	Platinum extraction	Liquid	308–312	US2024262853A1
Calix[4]pyrrole polymer	Lithium extraction	Liquid	290, 314 and 315	US20230072446A1
CB[7]	Separation	Liquid	318–321	WO2021124219A1

However the translational story for each of these supramolecular innovations differs,^153^ to continue to translate these innovations for commercial use, the teams seeking to develop these technologies will require the necessary infrastructure and support (both financial and knowledge based) to enable success. To our knowledge no venture capital or angel investor group exists with a primary interest to support the translation of supramolecular innovations, although a number of these groups do exist to support the translation of innovations within other fields, such as Synthetic Biology. Therefore, we believe that to continue to observe an increase in the number of supramolecular innovations to be successfully commercialised, specialist investment groups should be founded to facilitate the movement of these supramolecular technologies out of the labs and into products to serve humanity.

## Author contributions

DEB, EF, AL, MS, AMW: analysis, data collection, validation, writing – original draft, review & editing; PAG, CJEH, GTW, JRH: conceptualization, investigation, analysis, data collection, validation, funding acquisition, project administration, supervision, writing – original draft, review & editing.

## Conflicts of interest

PAG is a member of Chemical Society Reviews Advisory Board and is a former Chair of the Editorial Board.

## Data Availability

No primary research results, software or code have been included and no new data were generated or analysed as part of this review.
